# Pressure-Induced Reduction of Dicyanamide by Samarium(II) in a Coordination Polymer

**DOI:** 10.1021/acs.inorgchem.6c00425

**Published:** 2026-03-30

**Authors:** Hannah B. Wineinger, Tyler W. Hines, Kacy N. Mendoza, Thayalan Rajeshkumar, Nicholas B. Beck, Laurent Maron, Joseph M. Sperling, Thomas E. Albrecht

**Affiliations:** † Department of Chemistry and Nuclear Science & Engineering Center, 3557 Colorado School of Mines, Golden, Colorado 80401, United States; ‡ LPCNO, 27091 INSA Toulouse, CNRS, Université de Toulouse, 31077 Toulouse, France

## Abstract

A samarium­(II) coordination polymer, [Sm­(2.2.2-cryptand)­(dca)]­I (dca^−^ = dicyanamide), has been prepared from the reaction of SmI_2_ with tetrabutylammonium dicyanamide and 2.2.2-cryptand. The structure consists of [Sm­(2.2.2-cryptand)]^2+^ cations bridged by dicyanamide to form corrugated 1D chains. Single crystals of this compound have been studied spectroscopically using UV–vis–NIR and Raman spectroscopy as a function of pressure to reveal a pressure-induced two-electron reduction of dicyanamide by Sm^2+^ to cyanide and cyanamide and Sm^3+^ between 5 and 8 GPa. High-pressure single-crystal diffraction studies reveal a phase change at 2.6 ± 0.1 GPa, where a polymorph exhibiting a smaller Sm^2+^···N≡C bond angle and shorter Sm^2+^···C distance was observed. These observations provide mechanistic insight into the reduction of dicyanamide into cyanide and cyanamide by pressurized samarium­(II).

## Graphical abstract

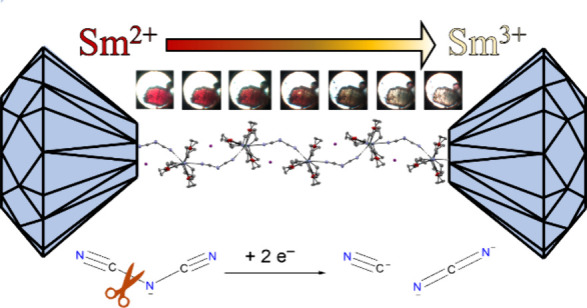

## Introduction

Samarium­(II) is a commonly used, one-electron reductant with many applications in organic synthesis^1^ with a demonstrated efficacy for a wide range of purposes, including magnetism^2,3^ and photochemical applications^4^ as well as pressure^5^ and temperature sensing.^6^ Compared with a 3+/2+ couple of −1.50 ± 0.01 V (vs SCE),^7^ Sm^2+^ is a stronger reductant than Eu^3+/2+^ (−0.34 ± 0.01 V)^7^ or Yb^3+/2+^ (−1.18 ± 0.01 V)^7^ and possesses a 4f^6^ electron configuration capable of 5d → 4f and/or 4f → 4f emission depending on the coordination environment and temperature.^8–11^ Samarium­(II) complexes with a broad array of ligands have been prepared, particularly substituted cyclopentadienyl anions including (Me_3_Si)­C_5_H_4_^–^,^12,13^ (Me_3_Si)_2_C_5_H_4_^–^,^14–16^ C_5_Me_5_^–^,^17^ Cp^*i*^Pr_5_^–^,^18,19^ and many others,^20–22^ various pyrrolides,^23,24^ (un)­substituted tris­(pyrazolylborates),^25–27^ crown/triflates,^28^ and crown/pseudohalides such as O=C=P^–^,^29^ to name a few. However, coordination polymers containing Sm^2+^ and no other cations are few in number despite valence tautomerism being exhibited by SmI_2_(pyrazine)_3_.^30^

Inspired by the beneficial properties of other pseudohalides that include improved solubility in polar solvents and ambidenticity (particularly thiocyanate engaging in N,S-bridging with Eu^2+^),^31^ we have expanded these studies to include the more complex pseudohalide, dicyanamide (dca^–^). Dicyanamide is a coordinating anion of choice for forming a wide variety of extended structures due to its bridging flexibility, and thorough research has been performed on transition metal dicyanamide metal–organic frameworks.^32^ Although a Sm^2+^/dca^–^ coordination polymer has not been previously observed in the solid state, several trivalent lanthanide coordination polymers and metal–organic frameworks are known, including Ln^III^(dca)_3_ (Ln = La, Ce, Pr, Nd, Sm, and Eu)^33^ and Ln^III^(H_2_O)_2_(dca)_3_ (Ln = Tb, Eu)^34^ 3D frameworks as well as heteroleptic discrete molecules^35,36^ and one 1D polymer.^36^ In these lanthanide­(III) compounds, dca^–^ is coordinated as a capping or bridging anion, bridging two (μ) or three (μ_3_) metal centers via the two cyano functional groups or both cyano groups and the amide nitrogen, respectively. In solution-based electrochemical studies, an unknown but possibly dca^–^-based Sm^2+^ complex has been shown to be an intermediate in the two-step electrodeposition process of Sm^3+^ to Sm^0^ in 1-butyl-1-methylpyrrolidinium dicyanamide,^37,38^ but further study on low-valent samarium compounds with dca^–^ has not been performed. Herein, we present the synthesis and characterization of [Sm­(2.2.2-cryptand)­(dca)]I as well as high-pressure studies on this compound that show the Sm^2+^ reduction of dicyanamide to cyanamide and cyanide.

## Experimental Section

All chemical manipulations were performed using standard argon Schlenk line and glovebox techniques. Samarium­(II) iodide (≥99.9%), sodium dicyanamide (96%), and 2.2.2-cryptand (≥99%) were all purchased from Sigma-Aldrich, and tetrabutylammonium chloride (≥97%) was purchased from Fluka. All chemicals were used as received without further purification. Acetonitrile (99.8%) and diethyl ether (≥99.0%) were also purchased from Sigma-Aldrich and loaded into a Vigor solvent purification system prior to use. Instrumentation details can be found in the Supporting Information.

### Synthesis of [Sm(2.2.2)­(μ-dca)]­I

In an argon glovebox, a suspension of tetrabutylammonium chloride (13.8 mg, 0.0500 mmol) and sodium dicyanamide (4.4 mg, 0.0494 mmol) in 1 mL of acetonitrile was stirred at 50 °C for 1 h in a 7 mL screw-top vial. The suspension was centrifuged to remove undissolved starting materials and precipitated sodium chloride, and the supernatant was used to dissolve 2.2.2-cryptand (9.3 mg, 0.0247 mmol). The colorless solution was transferred into another vial containing samarium diiodide (10.0 mg, 0.0247 mmol) to produce a red solution and some brown precipitate. This mixture was centrifuged, and the red supernatant was transferred into a fresh 7 mL vial before 5 drops of diethyl ether were added and the reaction was placed in a −35 °C freezer overnight. In the subsequent 2 days, 5 drops of diethyl ether were added to the reaction each night before it was replaced in the freezer. On the third day, the solution was decanted from the crystals, and the crystals were washed thrice with diethyl ether. This procedure was replicated six times, and in two of the six trials, crystallization was first observed overnight, while the other four had crystals on after the second night. The red block crystals were characterized by single-crystal X-ray diffraction as [Sm­(2.2.2-cryptand)­(μ-dca)]I despite varying widely in size, and the average reaction yield was 62.4% (11.1 mg, 0.0154 mmol). Yields from all reactions were consolidated into one vial prior to characterization to ensure consistency across the individual samples. See Figure S1 for photographs of the reaction progression and Figure S12 for ^1^H NMR.

**Caution!** Samarium­(II) diiodide (SmI_2_; CAS#: 32248-43-4) is a strong reducing agent, which reacts violently with some organic materials and must be handled with extreme care. One possible reduction product of cyano-functionalized chemicals such as sodium dicyanamide is cyanide, known for its acute toxicity (category 1) and tendency to form toxic, gaseous HCN.

## Results and Discussion

### Synthesis

[Sm­(2.2.2-cryptand)­(dca)]I was synthesized by metathesis in acetonitrile using in situ tetrabutylammonium dicyanamide and samarium diiodide in the presence of 2.2.2-cryptand, resulting in the formation of insoluble brown solids and a red-brown solution. After centrifugation, the red-brown supernatant was stored in the freezer (−35 °C) and daily additions of 5 drops of diethyl ether were performed. Crystallization occurred within 2 or 3 days. Red, block-shaped crystals were observed, and X-ray diffraction of a single crystal resulted in the formulation of [Sm­(2.2.2-cryptand)­(dca)]­I. To assess phase purity, powder X-ray diffraction was performed and can be found in the Supporting Information along with the ^1^H NMR spectrum (Figures S5 and S12). Further crystallographic details can be found in Tables S1–S7.

### X-ray Diffraction

[Sm­(2.2.2-cryptand)­(dca)]I crystallizes in the monoclinic space group *P*2_1_/*n*, forming corrugated 1D chains where each 10-coordinate Sm^2+^ sphenocorona^39^ center is encapsulated in a 2.2.2-cryptand molecule and bridged by dicyanamide anions at two sides (see Figure 1). The [Sm­(2.2.2-cryptand)­(dca)]^+^ chains are charge-balanced by one noncoordinating I^–^ anion in addition to one coordinating dicyanamide anion per Sm^2+^ center. The intrachain Sm^2+^–Sm^2+^ distance is 9.0932(6) Å, and the interchain interactions consist of short contacts between aliphatic cryptands and the sp^3^ carbons of dicyanamide anions (C–H···C) or iodide anions (C–H···I^–^···H–C). When placed in a diamond anvil cell under moderate pressure (1.5 ± 0.1 GPa; Figure S2), the same ambient pressure *P*2_1_/*n* phase of [Sm­(2.2.2-cryptand)­(dca)]­I is found.

**Figure 1:**
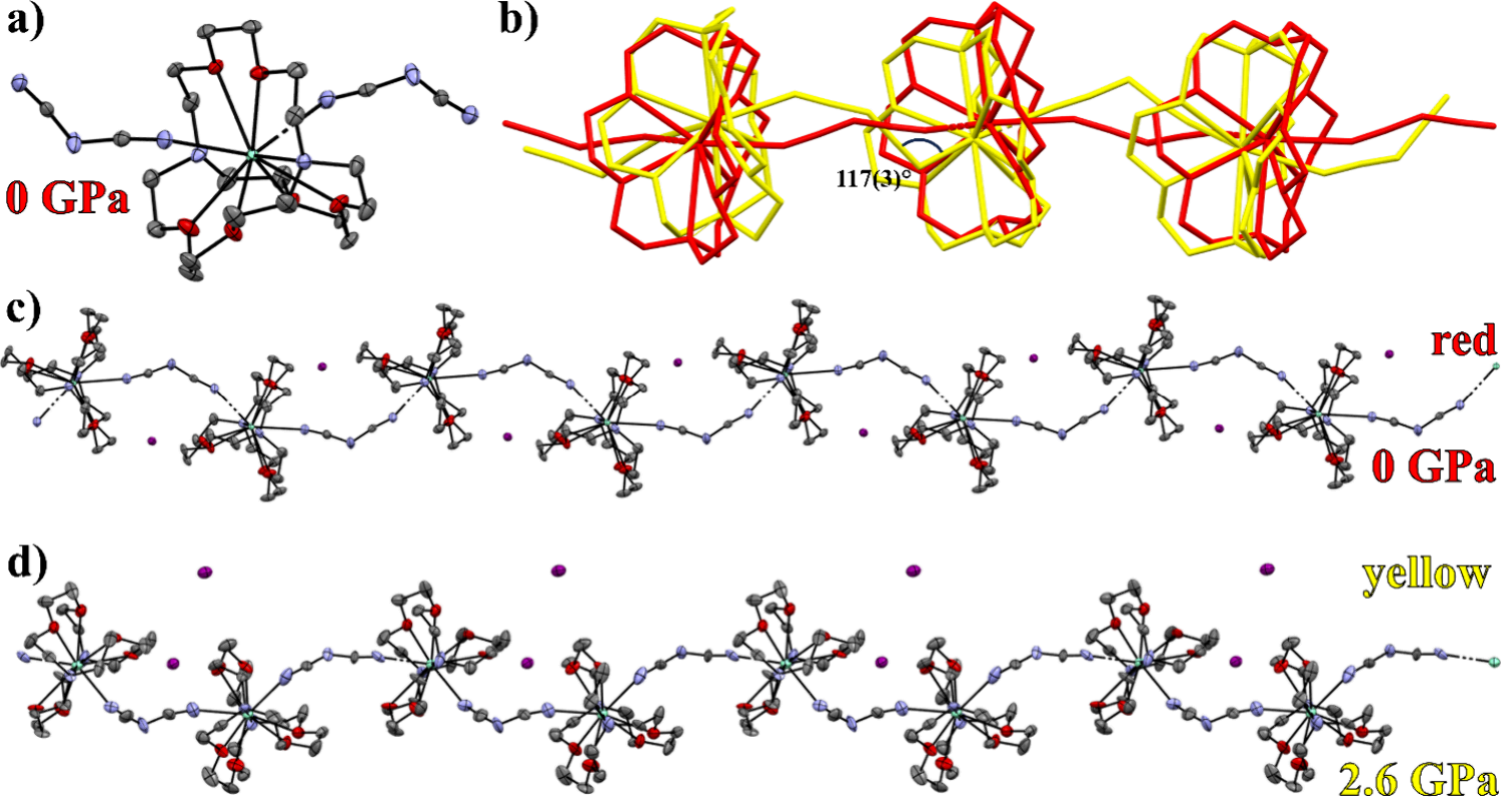
Structure of [Sm­(2.2.2-cryptand)­(dca)]I showing (a) the coordination environment of Sm^2+^ at ambient pressure and 100 K; (b) an overlay of [Sm­(2.2.2-cryptand)­(dca)]I at ambient pressure and 100 K (*P*2_1_/*n*; red) and 2.6 GPa (*Pna*2_1_; yellow); (c) 1D polymeric extended structure of ambient pressure, 100 K [Sm­(2.2.2-cryptand)­(dca)]­I (*P*2_1_/*n*); and (d) 1D polymeric extended structure of 2.6 GPa [Sm­(2.2.2-cryptand)­(dca)]I (*Pna*2_1_). Hydrogens are omitted for clarity. Excluding the overlay in (b), ellipsoids are at the 50% probability level, and Sm^2+^ atoms are represented as light green, carbon as gray, nitrogen as blue, oxygen as red, and iodide as purple.

However, at an applied pressure of 2.6 ± 0.1 GPa (Figure S3), a phase change to the higher symmetry orthorhombic space group *Pna*2_1_ is observed. In this polymorph, two [Sm­(2.2.2-cryptand)]^2+^ complex cations are found in the asymmetric unit, each with a crystallographically independent iodide and a dca^–^ anion. The Sm^2+^···2.2.2-cryptand bond lengths are within the same range for both phases as compared with the ambient pressure structure; however, some Sm^2+^···N_dca_ bonds are observably shorter in the *Pna*2_1_ phase (2.56(3)–2.66(2) Å) than in the ambient pressure *P*2_1_/*n* phase (2.645(3)–2.668(3) Å).

The most striking differences are found in the bridging dicyanamide anions: where the *P*2_1_/*n* phase exhibits a tilted “end on” coordination of dicyanamide with Sm^2+^···N≡C angles between 155 and 160°, the two dicyanamides in the *Pna*2_1_ phase feature one canted and one straightened dicyanamide. The canted dicyanamide in *Pna*2_1_ results in a Sm^2+^···N≡C angle and Sm^2+^···nitrile carbon distances of 117(3)° and 3.26(4) Å on one side and 144(3)° and 3.67(2) Å on the other. The other, “straightened”, dicyanamide anion in *Pna*2_1_ demonstrates more linear coordination than observed in the *P*2_1_/*n* phase, where these values are 157(3)° and 3.71(3) Å for one side and 171(3)° and 3.79(3) Å for the other. The canted dicyanamide anion exhibits a dramatic shortening of the distance between Sm^2+^ and the nitrile carbon at 3.26(4) Å. The shortest distance between these two atoms in any other measurement is 3.70(3) Å from the *P*2_1_/*n* phase measured at 1.5 ± 0.1 GPa. This abrupt change in structure correlates well with the observed reactivity discussed below and may shed some light on the mechanism by which dicyanamide is reduced, particularly if an interaction between Sm^2+^ and a dicyanamide carbon is required for reduction to cyanide and cyanamide.

At higher applied pressures, crystals of [Sm­(2.2.2-cryptand)­(dca)]­I become yellow to colorless and amorphous, showing a few smeared reflections at 3.9 ± 0.2 GPa (Figure S4). This loss of crystallinity is expected, given that the suspected redox reaction should result in a samarium­(III) phase with stoichiometric equivalents of cyanide, cyanamide, and dicyanamide and no obvious coordination preference of these flexible ambidentate ligands.

### Spectroscopy

The solid-state and solution-phase UV–vis–NIR of [Sm­(2.2.2-cryptand)­(dca)]I exhibit broad, intense 4f → 5d transitions typical of samarium­(II) in the UV to visible region, with resolvable peaks at roughly 490 and 550 nm (see Figure S6). To utilize this unique opportunity to study Sm^2+^ coordination polymers, [Sm­(2.2.2-cryptand)­(dca)]I was placed in a diamond-anvil cell with a ruby internal standard, and the pressure increased incrementally while obtaining the corresponding solid-state UV–vis–NIR and Raman spectra. As shown in Figure 2, as the pressure was increased, the intensity of the 4f → 5d transitions decreased and the highly pigmented Sm^2+^ crystals changed from red to brown to pale yellow. At pressures of 5–8 GPa, the 4f → 5d transitions completely disappear, demonstrating a complete oxidation state change from Sm^2+^ to Sm^3+^ induced by the application of pressure.

**Figure 2:**
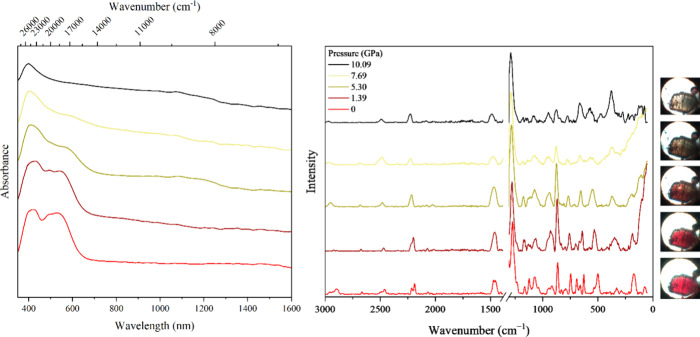
High-pressure UV–vis–NIR and Raman spectroscopy of [Sm­(2.2.2-cryptand)­(dca)]­I. For all data taken, see Figures S8 and S10.

The corresponding reduction is most likely to be the cleavage of a C–N bond between a cyano group and the amide in dicyanamide rather than the aliphatic 2.2.2-cryptand (Scheme 1). This type of bond-breaking has been observed in molten cyanide salts, where dicyanamide is reduced by two electrons (from Sn, Zn, Cu, or Ni) to form cyanamide and cyanide.^40^ In the high-pressure Raman experiments conducted herein, only a few changes are observed with the application of pressure: an increase in intensity at 1290 cm^–1^ (C=N) and a merging of the separate peaks indicative of a cyano functional group (2230 cm^–1^) to form one broad peak (see Table S8). These features are consistent with previously observed shifts for cyanamide, where free cyanamide accounts for cyano groups and C–N single bonds (at 2264–2270 and 1055–1061 cm^–1^, respectively)^41,42^ and metal cyanamide salts exhibit C=N bonds at ν_s_ = 1241.5 and 1338 cm^–1^.^43,44^ In short, increasing pressure on [Sm­(2.2.2-cryptand)­(dca)]I results in the growth of the cyanamide-indicative C=N peak (1290 cm^–1^) concomitant with the decrease of 4f → 5d transitions in the UV–vis–NIR of Sm^2+^, demonstrating that the application of pressure on [Sm­(2.2.2-cryptand)­(dca)]I induces a two-electron reduction of a samarium­(II) dicyanamide complex to form a samarium­(III) cyanamide/cyanide compound by 8 GPa. Cyanide (NaCN or KCN at 2091–2078 cm^–1^)^45^ cannot be distinguished from cyanamide or dicyanamide by Raman spectroscopy in this case, though all are expected to be present following this pressure-induced redox reaction.

**Scheme 1:**
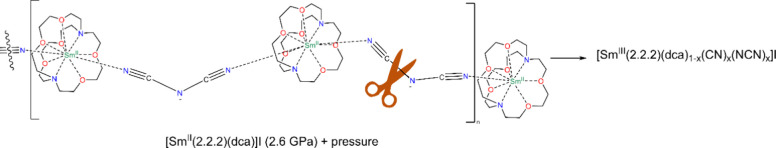
Reduction of Dicyanamide Starting from the *Pna*2_1_ Polymorph of [Sm­(2.2.2-cryptand)­(dca)]­I, Where *x* Is between 0 and 1

As a control experiment, a single crystal of [Sm­(2.2.2-cryptand)­(dca)]­I was loaded into a diamond anvil cell without applying more than 1 GPa of pressure, and the cell was left standing at ambient pressure for a time period similar to that observed during the high-pressure experiments (5 h). In this case, when additional pressure is not applied, no oxidation of [Sm­(2.2.2-cryptand)­(dca)]I or corresponding changes in the Raman spectra were observed (Figure S11), indicating that [Sm­(2.2.2-cryptand)­(dca)]I is reasonably stable in a diamond anvil cell setup if pressures >1 GPa are not applied. Similarly, crystals of [Sm­(2.2.2-cryptand)­(dca)]I placed under immersion oil at ambient pressure were measured as they degraded in air, and though oxidation does occur over a period of several days, the corresponding Raman spectra indicate this is not by reduction of dicyanamide into cyanide and cyanamide (Figures S7 and S9). Visual inspection of [Sm­(2.2.2-cryptand)­(dca)]I degraded under ambient pressure versus [Sm­(2.2.2-cryptand)­(dca)]I crystals oxidized by the application of pressure reveals that when pressure is applied to [Sm­(2.2.2-cryptand)­(dca)]I the majority of the crystal changes color simultaneously (Figure 2), while Figures S7 and S9 show oxidation primarily on the surface encroaching inward due to aerobic degradation. Experiments testing the reversibility of the pressure-induced redox reaction revealed that after crystals of [Sm­(2.2.2-cryptand)­(dca)]­I became colorless at ∼6 GPa, releasing the pressure in the diamond anvil cell did not restore the red coloration, indicating that the pressure-induced redox reaction is irreversible.

### Computations

DFT calculations (B3PW91 functional) were carried out on the system at ambient pressure and room temperature to further highlight that pressure induces the redox reaction between Sm^2+^ and dicyanamide. The structure of [Sm­(2.2.2-cryptand)­(dca)]­I was optimized in two different spin states as discussed in the SI, and as expected, no strong magnetic coupling exists between the two Sm­(II) centers. A natural bond orbital analysis was carried out in this complex. It is interesting to note that the dicyanamide anion, which is formally considered as having two terminal C≡N triple bonds and two internal C–N single bonds, shows polarization of the C–N single bonds toward N (60%). The Wiberg bond indexes, which are a diagnostic of covalency, appear to be 2.5 and 1.3 for the triple and single CN bonds, respectively, indicating that electron delocalization is occurring in the dicyanamide ligand. This is further illustrated by the HOMO–22 and HOMO–19 molecular orbitals (see Table S15), where a π-type delocalization involves all 5 atoms of the anion. Interestingly enough, the LUMO (see Figure 3) of the complex appears to involve the central C–N–C moiety with an antibonding interaction between the two N≡C π interactions and strong Sm^2+^–N bonding interactions.

**Figure 3:**
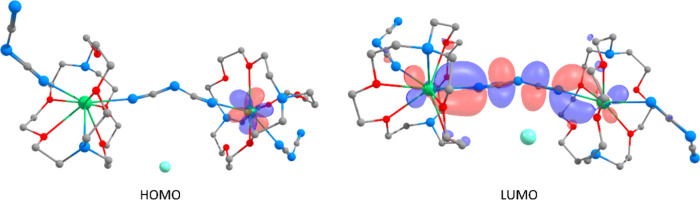
Computed HOMO (left) and LUMO (right) MOs (AMO) for [Sm­(2.2.2-cryptand)­(dca)]­I, *s* = 6. Hydrogen atoms are omitted for clarity, and samarium atoms are represented as green, iodide as aquamarine, oxygen as red, carbon as gray, and nitrogen as blue.

The central C–N bond breaking transition state (TS) was computed, and the associated barrier is predicted to be 41.2 kcal·mol^–1^ (Figure 4), indicating that the reaction is kinetically not possible under ambient pressure. At the TS, the central N–C bond is fully broken (2.09 vs 1.29 Å) and the two samarium centers are in the +3 oxidation state. This reaction is therefore a classical single electron transfer reduction of dicyanamide by two samarium­(II) centers. Although calculations including external pressure are difficult using a standard computational approach, it has been possible to estimate using frequency calculations that, at a pressure of 2.6 GPa, the barrier decreases to 22.8 kcal·mol^–1^, making the reaction kinetically accessible. The intrinsic reaction coordinate yields the final samarium­(III) complex in the presence of one cyanide and one cyanamide ligand. At room temperature and under ambient pressure, this reaction is endothermic by 9.5 kcal·mol^–1^, but again, estimating the effect of a 2.6 GPa pressure makes the reaction athermic (−0.6 kcal·mol^–1^).

**Figure 4:**
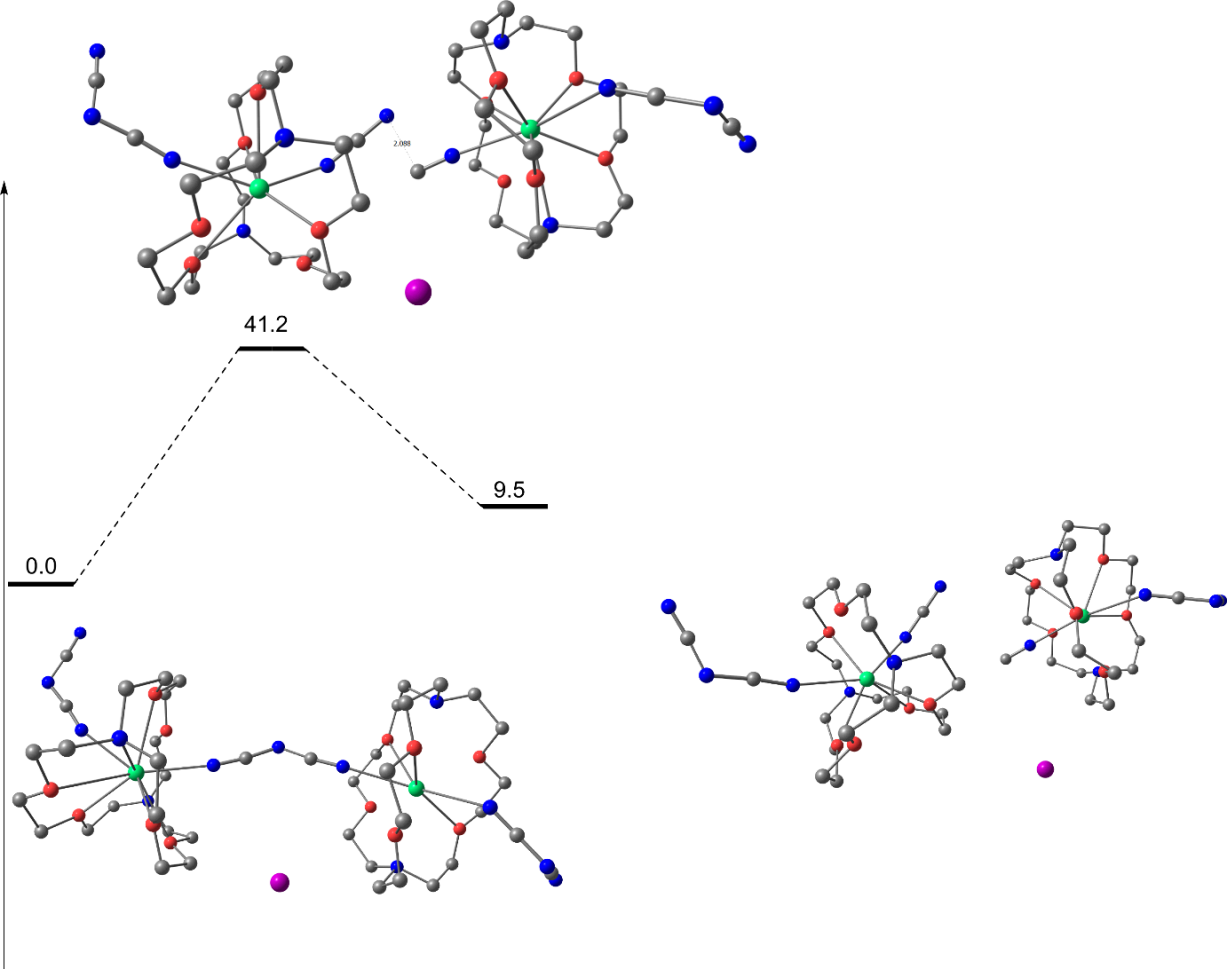
Computed enthalpy reaction pathway for the formation of cyanide and cyanamide complexes from [Sm­(2.2.2-cryptand)­(dca)]I at room temperature and under ambient pressure.

## Conclusion

In summary, the samarium­(II) coordination polymer [Sm­(2.2.2-cryptand)­(dca)]­I was synthesized and characterized by using single-crystal X-ray diffraction and UV–vis–NIR and Raman spectroscopy. High-pressure experiments induced the two-electron reduction of dicyanamide into cyanide and cyanamide using Sm^2+^, a mechanistic hypothesis supported by high-pressure Raman spectroscopic measurements showing cyanamide formation as a function of increasing pressure until 5–8 GPa, at which point the total oxidation of Sm^2+^ to Sm^3+^ occurred as determined by UV–vis–NIR spectroscopy.

## Supplementary Material



## Data Availability

All data generated or analyzed during this study are included in this published article (and its Supporting Information files), and crystallographic structures can be accessed via the Cambridge Crystallographic Data Centre using accession codes 2515480, 2515371, and 2515372.

## Notes

Pictures of reaction progression, solid-state and solution phase UV–vis–NIR spectroscopy, crystallographic tables, powder diffractograms, Raman and ^1^H NMR spectra, all high-pressure experiments, and computational details (PDF)
