# Supplementary material for: Pressure-Induced Reduction of Dicyanamide by Samarium(II) in a Coordination Polymer
Source: Inorg Chem. 2026 Mar 30;65(14):7974–80. doi: 10.1021/acs.inorgchem.6c00425 (PMC13080990; doi:10.1021/acs.inorgchem.6c00425)
Supplement: Supplementary file 1 [file ic6c00425_si_001.pdf]

Pressure-Induced Reduction of Dicyanamide by Samarium(II) in a Coordination Polymer

Hannah B. Wineinger,[a] *Tyler W. Hines,*[a] *Kacy N. Mendoza,*[a] *Thayalan Rajeshkumar,*[b] *Nicholas B. Beck,*[a] *Laurent Maron,*[b,*] *Joseph M. Sperling,*[a,*] *Thomas E. Albrecht*[a,*]

[a]Department of Chemistry and Nuclear Science & Engineering Center, Colorado School of Mines, Golden, CO 80401, USA

[b]Université de Toulouse, INSA Toulouse, CNRS, LPCNO, 31077 Toulouse, France

*Corresponding author emails: laurent.maron@irsamc.ups-tlse.fr, jsperling@mines.edu; thomas.albrecht@mines.edu

Table of Contents

Instrumentation	S2
Synthetic Details	S4
Crystallographic Tables	S5
Powder X-Ray Diffraction	S13
UV-vis-NIR Spectroscopy	S14
Raman Spectroscopy	S17
Diamond Anvil Cell Control Experiment	S19
^{1}H-NMR Spectroscopy	S20
Computations	S21
References	S46

Instrumentation

X-Ray Diffraction. Ambient pressure single crystal X-ray diffraction was performed using a three axis Bruker D8 Quest diffractometer equipped with a sealed IμS Mo K_α X-ray source (λ = 0.71073 Å) and a photon III detector. High pressure single crystal X-ray diffraction was performed on a Bruker Venture diffractometer using a sealed Ag X-ray source (λ = 0.56086 Å) at room temperature. Single crystals of [Sm(2.2.2-cryptand)(dca)]I were placed on a glass slide under immersion oil and then removed from the glovebox. Individual crystals were then evaluated visually under a microscope before selecting one for diffraction experiments and mounting on the diffractometer, either on a 75 μm MiTiGen cryoloop or inside a diamond anvil cell, respectively. Reflections were indexed and integrated using Bruker's APEX4 software,[1] and the appropriate space group was determined using xprep.[2] The solved structure was refined through the Olex2 GUI using the XT program from the SHELX suite.[3-5]

Powder X-ray measurements were taken on a Bruker D2 Phaser in the Bragg-Brentano geometry using a Cu X-ray source (λ = 1.54184 Å) and a LYNXEYE_XE_T (1D) detector at room temperature. Diffractograms were collected with an approximate step size of 0.024°/step in the region of 5 – 30° 2θ with a measurement time of 1 s per step. The sample of [Sm(2.2.2)(μ-dca)]I was briefly ground up with mortar and pestle inside the argon glovebox before transferring roughly 20 mg of material as a red powder onto a silicon wafer and enclosing the sample using the screw-top knife-edge dome.

Spectroscopy. Solution phase UV-vis-NIR spectra were taken on the Cary 6000*i* from Agilent Technologies using Starna quartz cuvettes. The solution was prepared inside the argon glovebox by dissolving the appropriate amount of [Sm(2.2.2-cryptand)(dca)]I in 2 mL of acetonitrile using a micropipette. The prepared solution was then transferred into a Starna quartz cuvette and tightly

capped. The sample was immediately measured after removal from the glovebox. The spectrum was taken between 200 – 1000 nm with a step size of 0.250 nm and a measurement rate of 75 nm/min. Solid-state UV-vis-NIR spectra were collected using the Craic Technologies Microspectrophotometer equipped with a Xe lamp (75 W). Absorbance measurements were taken on individual [Sm(2.2.2-cryptand)(dca)]I crystals on a glass slide under normal atmospheric conditions.

Raman spectra were collected using a Bruker Senterra II using a 785 nm laser (grating 400) with an aperture of 50×1000 μm. Crystals were placed on a glass slide under a 20× objective prior to measurement. The ^{1}H-NMR spectrum was measured on JEOL (ECA-500) 500 MHz liquids only NMR instrument, where the sample was measured in CD_3CN using internal standard trace solvent as the calibrant.

High-Pressure Diamond Anvil Cell. A 200 μm thick steel gasket was indented to approximately 70 μm and then a hole was drilled through the center of the indentation using a Boehler μDriller electric drill. The gasket was set in a DACTools SSDAC80 and a single crystal of [Sm(2.2.2-cryptand)(dca)]I was loaded inside the gasket hole along with a few ruby spheres for measurement of pressure inside the cell. A drop of polydimethylsiloxane was added inside the gasket hole as the pressure medium and the diamond anvil cell was sealed. Pressure was measured during the experiments through the positions of the fluorescence peak of the rubies around 694 nm when excited by a 546 nm laser on the Craic Technologies microspectrophotometer. Solid-state UV-Vis-NIR data on [Sm(2.2.2-cryptand)(dca)]I was collected at each pressure with the same microspectrophotometer. Raman spectra were also collected at each pressure with a Bruker Senterra II using a 785 nm laser.

Synthetic Details

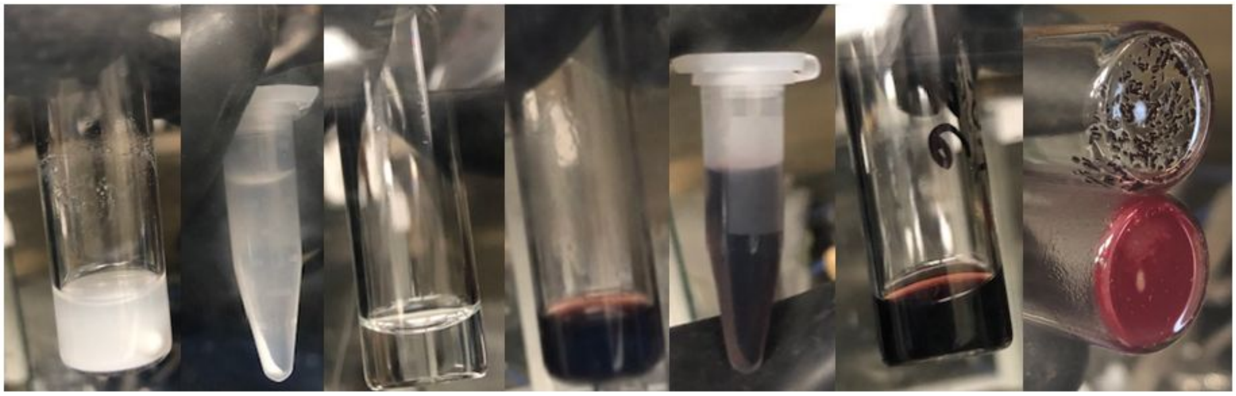

Figure S1. Progression of the reaction resulting in [Sm(2.2.2-cryptand)(dca)]I. From left to right: an image of the mixture of tetrabutylammonium chloride and sodium dicyanamide in acetonitrile with stir bar; the centrifuged mixture with undissolved starting materials and sodium chloride; the solution of *in situ* tetrabutylammonium dicyanamide with 2.2.2-cryptand; a mixture of *in situ* tetrabutylammonium dicyanamide with 2.2.2-cryptand and samarium diiodide; centrifugation of the previous mixture with brown solid and red reaction solution; the red reaction solution with 5 drops of diethyl ether; crystals from two different trials of this same procedure, highlighting the wide range of crystal sizes obtained.

Crystallographic Tables

Table S1. Comparison of all Sm^{n+} and Eu^{n+} dca^{-} single crystals (n = 2, 3).

**Compound**	**dca^{-} Bridging[a]**	**M^{n+} – N (nitrile) / Å**	**M^{n+} – N (amide) / Å**	**Ancillary Ligand Bond Lengths / Å**	**Polymer Type (or Discrete)**	**Ref**
$Sm^{III}(dca)_3$	μ_3	2.50, 2.57	2.65	–	3D	6
$[Sm^{III}(phen)_2(H_2O)_3(dca)_2]dca\cdot phen$[b]	μ	2.478(3), 2.500(3),	–	2.433(2)-2.443(2) (H_2O) 2.635(2)-2.720(2) (phen)[b]	discrete monomer	7
$[Sm^{III}(bipy)_2(H_2O)(dca)_3]_{1\infty}$[c]	μ	2.508, 2.516	–	2.456 (H_2O) 2.633, 2.691 (bipy)[c]	1D	7
$[Sm^{II}(2.2.2\text{-cryptand})(dca)]I$	μ	2.645(3), 2.668(3)	–	2.706(2)-2.773(2) (Sm^{2+} – O, 2.2.2)[d] 2.980(3)-2.983(3) (Sm^{2+} – N, 2.2.2)[d]	1D	this work
$Eu^{III}(dca)_3$	μ_3	2.38, 2.50	2.72, 2.88	–	3D	6
$Eu^{III}(H_2O)_2(dca)_3$	μ	2.5(1)-2.62(2)	–	2.37(5), 2.41(7) (H_2O)	3D	8
$[C_2mim][Eu^{III}(H_2O)_4(dca)_4]$[d]	capping	2.48(1)-2.51(2)	–	2.32(1)-2.41(1) (H_2O)	discrete monomer	9

[a]Bridging dca^{-} are classified as capping, meaning no bridging; μ, bridging two metal centers through the two cyano arms of dicyanamide; and μ_3 bridging three metal centers, one through each nitrogen in dicyanamide; [b]phen is a abbreviation of phenanthroline; [c]bipy is an abbreviation of 2,2'-bipyridine; [d][C_2mim] is the complex cation 1-ethyl-3-methylimdazolium.

Table S2. Crystal data and structure refinement for [Sm(2.2.2-cryptand)(dca)]I (ambient pressure).

Formula	$C_{20}H_{36}IN_5O_6Sm$
Molar Mass / g·mol^{-1}	719.79
Color	red
Crystal habit	block
Temperature / K	100
Crystal system	monoclinic
Space group	$P2_1/n$
a / Å	11.3128(4)
b / Å	17.5477(6)
c / Å	13.4348(4)
α / °	90
β / °	93.3830(10)
γ / °	90
Volume / Å^3	2662.34(15)
Z	4
$\rho_{calc.}$ / g·cm^{-3}	1.796
μ / mm^{-1}	3.407
F(000)	1416
Crystal size / mm^3	0.051 × 0.05 × 0.049
Radiation	Mo K_α (λ = 0.71073 Å)
2Θ range / °	3.822 to 55.892
Index ranges	$-14 \le h \le 14$, $-23 \le k \le 23$, $-17 \le l \le 17$
Reflections collected	100318
Independent reflections	6379 [R_{int} = 0.0829, R_{sigma} = 0.0294]
Data/restraints/parameters	6379/0/298
Goodness-of-fit on F^2	1.06
Final R indexes [I ≥ 2σ(I)]	R_1 = 0.0278, wR_2 = 0.0486
Final R indexes [all data]	R_1 = 0.0400, wR_2 = 0.0530
$\Delta\rho_{max}$, $\Delta\rho_{min}$ / e·Å^{-3}	1.37; −0.57

Table S3. Bond lengths of [Sm(2.2.2-cryptand)(dca)]I (ambient pressure).

Atom	Atom	Length/Å	Atom	Atom	Length/Å
Sm1	O3	2.762(2)	O6	C17	1.434(4)
Sm1	O4	2.727(2)	N5	C20	1.151(5)
Sm1	O1	2.758(2)	N1	C7	1.477(4)
Sm1	O2	2.706(2)	N1	C1	1.480(4)
Sm1	O5	2.708(2)	N1	C13	1.477(5)
Sm1	O6	2.773(2)	N2	C12	1.470(4)
Sm1	N5[a]	2.667(3)	N2	C6	1.468(4)
Sm1	N1	2.983(3)	N2	C18	1.484(4)
Sm1	N2	2.979(3)	C20	N4	1.308(4)
Sm1	N3	2.645(3)	N3	C19	1.157(5)
O3	C8	1.431(4)	N4	C19	1.304(5)
O3	C9	1.423(4)	C8	C7	1.505(5)
O4	C10	1.428(4)	C12	C11	1.494(5)
O4	C11	1.425(4)	C9	C10	1.494(5)
O1	C2	1.443(4)	C4	C3	1.491(5)
O1	C3	1.430(4)	C6	C5	1.508(5)
O2	C4	1.430(4)	C1	C2	1.495(5)
O2	C5	1.420(4)	C16	C15	1.486(6)
O5	C14	1.409(4)	C17	C18	1.503(5)
O5	C15	1.432(4)	C14	C13	1.503(5)
O6	C16	1.435(4)			

[a]3/2−x, −1/2+y, 3/2−z

Table S4. Crystal data and structure refinement for [Sm(2.2.2-cryptand)(dca)]I (1.5 ± 0.1 GPa).

Formula	$C_{20}H_{36}IN_5O_6Sm$
Molar Mass / g·mol^{-1}	719.79
Color	red
Crystal habit	block
Temperature / K	296.15
Crystal system	monoclinic
Space group	$P2_1/n$
a / Å	11.3199(8)
b / Å	17.501(3)
c / Å	13.5843(10)
α / °	90
β / °	92.868(4)
γ / °	90
Volume / Å^3	2687.9(5)
Z	4
$\rho_{calc.}$ / g·cm^{-3}	1.779
μ / mm^{-1}	1.784
F(000)	1416
Crystal size / mm^3	0.05 × 0.05 × 0.01
Radiation	Ag K_α (λ = 0.56086 Å)
2Θ range / °	2.998 to 47.45
Index ranges	$-16 \leq h \leq 16$, $-8 \leq k \leq 8$, $-19 \leq l \leq 19$
Reflections collected	41232
Independent reflections	3202 [R_{int} = 0.2411, R_{sigma} = 0.1632]
Data/parameters/restraints	3202/43/298
Goodness-of-fit on F^2	1.028
Final R indexes [I ≥ 2σ(I)]	R_1 = 0.0669, wR_2 = 0.1642
Final R indexes [all data]	R_1 = 0.1618, wR_2 = 0.2099
$\Delta\rho_{max}$, $\Delta\rho_{min}$ / e·Å^{-3}	0.69; −0.61

Table S5. Bond lengths of [Sm(2.2.2-cryptand)(dca)]I (1.5 ± 0.1 GPa).

Atom	Atom	Length/Å	Atom	Atom	Length/Å
Sm1	O5	2.78(2)	N2	C6	1.42(3)
Sm1	N3	2.64(3)	N2	C18	1.54(6)
Sm1	O3	2.737(13)	O6	C16	1.44(3)
Sm1	O1	2.731(12)	O6	C17	1.40(2)
Sm1	O4	2.722(11)	O2	C5	1.40(2)
Sm1	N2	2.957(13)	O2	C4	1.40(3)
Sm1	O6	2.68(2)	C8	C7	1.45(4)
Sm1	O2	2.790(11)	N1	C1	1.48(5)
Sm1	N1	2.955(12)	N1	C7	1.46(2)
Sm1	N5[a]	2.61(4)	N1	C13	1.46(6)
O5	C14	1.45(2)	C16	C15	1.44(2)
O5	C15	1.33(6)	C2	C1	1.54(3)
N3	C19	1.17(6)	C20	N4	1.38(6)
C19	N4	1.21(6)	C20	N5	1.13(2)
O3	C8	1.40(2)	C12	C11	1.44(2)
O3	C9	1.40(3)	C6	C5	1.43(5)
O1	C2	1.390(15)	C3	C4	1.42(3)
O1	C3	1.400(18)	C14	C13	1.44(7)
O4	C11	1.45(2)	C9	C10	1.40(3)
O4	C10	1.423(19)	C17	C18	1.38(5)
N2	C12	1.45(4)			

[a]1/2−x, −1/2+y, 3/2−z

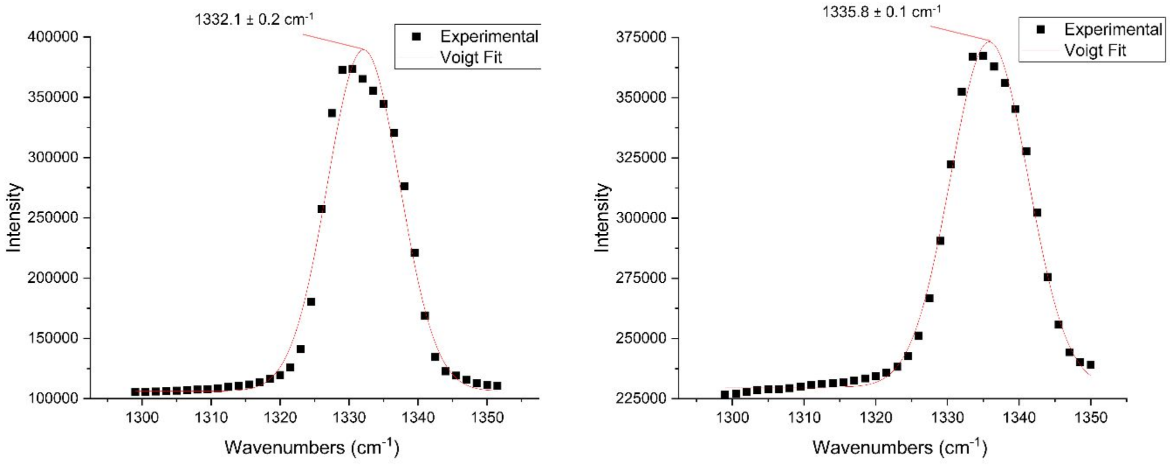

Figure S2. Raman spectra of the diamond anvil cell before (left) and after (right) the application of pressure for the 1.5 ± 0.1 GPa structure of [Sm(2.2.2-cryptand)(dca)]I. The values used to calculate the applied pressure have been labelled as data callouts on the associated diamond peak.

Table S6. Crystal data and structure refinement for [Sm(2.2.2-cryptand)(dca)]I (2.6 ± 0.1 GPa).

Formula	$C_{40}H_{72}I_2N_{10}O_{12}Sm_2$
Molar Mass / g·mol^{-1}	1439.57
Color	red
Crystal habit	block
Temperature / K	296.15
Crystal system	orthorhombic
Space group	$Pna2_1$
a / Å	32.625(5)
b / Å	10.347(2)
c / Å	13.015(3)
α / °	90
β / °	90
γ / °	90
Volume / Å^3	4394(2)
Z	4
$\rho_{calc.}$ / g·cm^{-3}	2.176
μ / mm^{-1}	2.183
F(000)	2832
Crystal size / mm^3	0.05 × 0.05 × 0.01
Radiation	Ag K_{α} (λ = 0.56086)
2Θ range / °	1.97 to 42.238
Index ranges	$-41 \leq h \leq 40$, $-11 \leq k \leq 11$, $-13 \leq l \leq 14$
Reflections collected	30636
Independent reflections	6896 [R_{int} = 0.1350, R_{sigma} = 0.1185]
Data/parameters/restraints	6896/115/596
Goodness-of-fit on F^2	1.045
Final R indexes [I ≥ 2σ(I)]	R_1 = 0.0734, wR_2 = 0.1741
Final R indexes [all data]	R_1 = 0.1187, wR_2 = 0.2035
$\Delta\rho_{max}$, $\Delta\rho_{min}$ / e·Å^{-3}	2.17; −1.90
Flack parameter	0.41(7)

Table S7. Bond lengths of [Sm(2.2.2-cryptand)(dca)]I (2.6 ± 0.1 GPa).

Atom	Atom	Length/Å	Atom	Atom	Length/Å
Sm1	O1	2.93(2)	O6	C17	1.42(4)
Sm1	O5	2.71(2)	O6	C16	1.42(4)
Sm1	O2	2.64(2)	N6	C33	1.46(4)
Sm1	O4	2.69(2)	N6	C27	1.51(4)
Sm1	N3	2.56(3)	N6	C21	1.52(4)
Sm1	O3	2.55(2)	O8	C24	1.40(4)
Sm1	O6	2.689(18)	O8	C25	1.41(4)
Sm1	N2	2.76(3)	N8	C39	1.22(4)
Sm1	N1	2.91(2)	C7	N1	1.43(3)
Sm1	N10[a]	2.65(3)	C7	C8	1.54(5)
Sm2	O12	2.71(2)	C10	C9	1.49(4)
Sm2	O11	2.67(2)	C19	N4	1.31(5)
Sm2	O9	2.69(2)	N9	C39	1.30(4)
Sm2	O10	2.72(3)	N9	C40	1.33(3)
Sm2	O7	2.54(2)	C3	C4	1.52(5)
Sm2	N6	2.93(3)	C36	C35	1.51(5)
Sm2	O8	2.62(3)	N2	C6	1.51(5)
Sm2	N8	2.55(3)	N2	C18	1.49(3)
Sm2	N5	2.66(2)	N2	C12	1.49(5)
Sm2	N7	2.90(3)	C17	C18	1.46(5)
O1	C3	1.41(4)	C28	C27	1.51(4)
O1	C2	1.44(4)	C37	C38	1.47(4)
O12	C36	1.44(3)	N5	C20	1.16(3)
O12	C37	1.40(5)	C6	C5	1.44(5)
O11	C34	1.42(4)	C2	C1	1.49(4)
O11	C35	1.41(3)	N1	C13	1.47(3)
O9	C28	1.45(4)	N1	C1	1.45(4)
O9	C29	1.34(5)	C13	C14	1.49(7)
O5	C15	1.42(3)	C20	N4	1.33(4)
O5	C14	1.42(6)	C16	C15	1.51(5)
O2	C5	1.44(4)	C34	C33	1.48(5)
O2	C4	1.40(3)	C32	N7	1.45(4)
O4	C10	1.46(4)	C32	C31	1.43(6)
O4	C11	1.45(4)	C24	C23	1.59(5)
O10	C31	1.44(4)	N7	C38	1.46(3)
O10	C30	1.43(4)	N7	C26	1.51(5)
N3	C19	1.17(5)	C29	C30	1.50(6)
O7	C22	1.41(4)	N10	C40	1.15(4)
O7	C23	1.39(4)	C22	C21	1.53(5)
O3	C9	1.42(4)	C25	C26	1.52(6)
O3	C8	1.47(4)	C11	C12	1.63(5)

[a]−1/2+x, 1/2−y, z

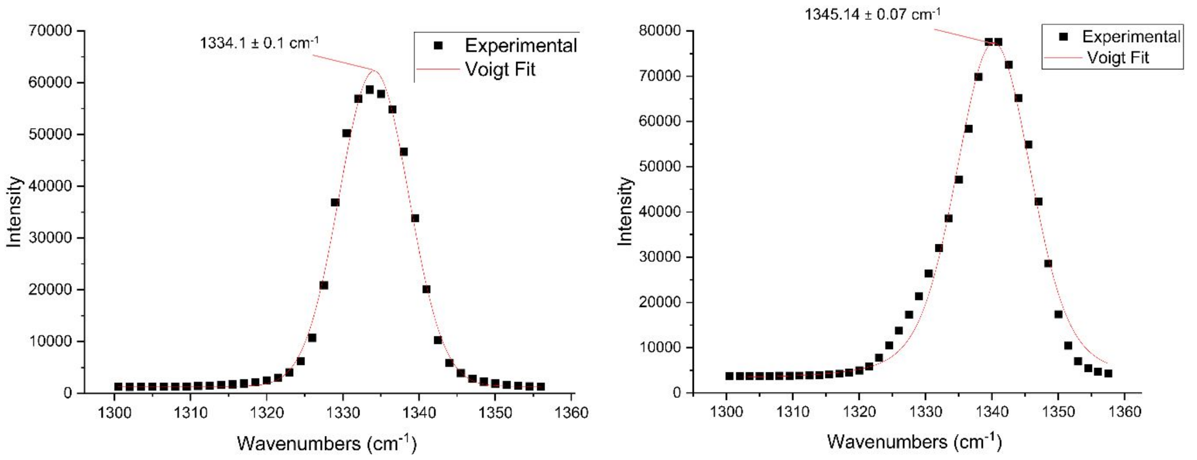

Figure S3. Raman spectra of the diamond anvil cell before (left) and after (right) the application of pressure for the 2.6 ± 0.1 GPa phase of [Sm(2.2.2-cryptand)(dca)]I. The values used to calculate the applied pressure have been labelled as data callouts on the associated diamond peak.

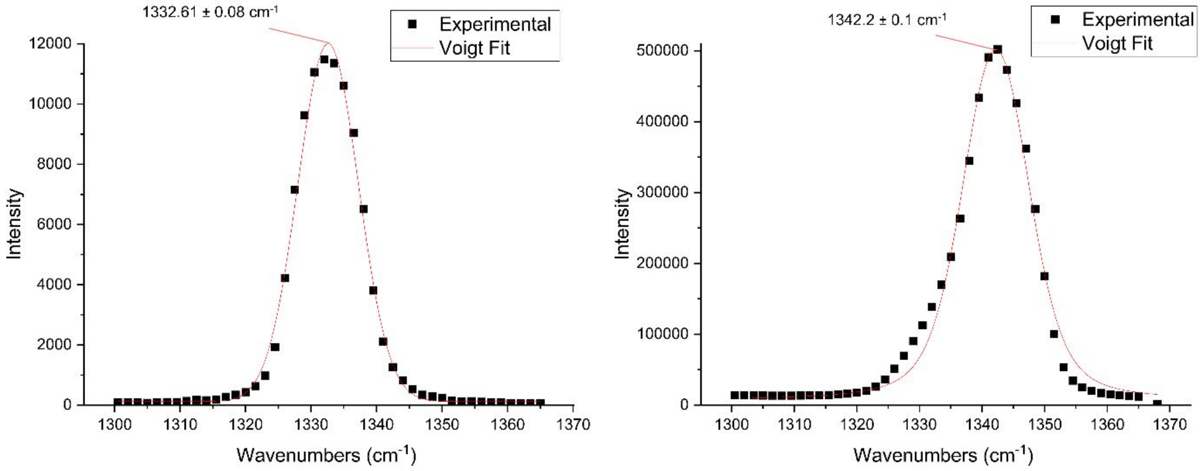

Figure S4. Raman spectra of the diamond anvil cell before (left) and after (right) the application of pressure of 3.9 ± 0.2 GPa on [Sm(2.2.2-cryptand)(dca)]I. Under this pressure, only a few reflections are observed and [Sm(2.2.2-cryptand)(dca)]I was amorphous. The values used to calculate the applied pressure have been labelled as data callouts on the associated diamond peak.

Powder X-Ray Diffraction

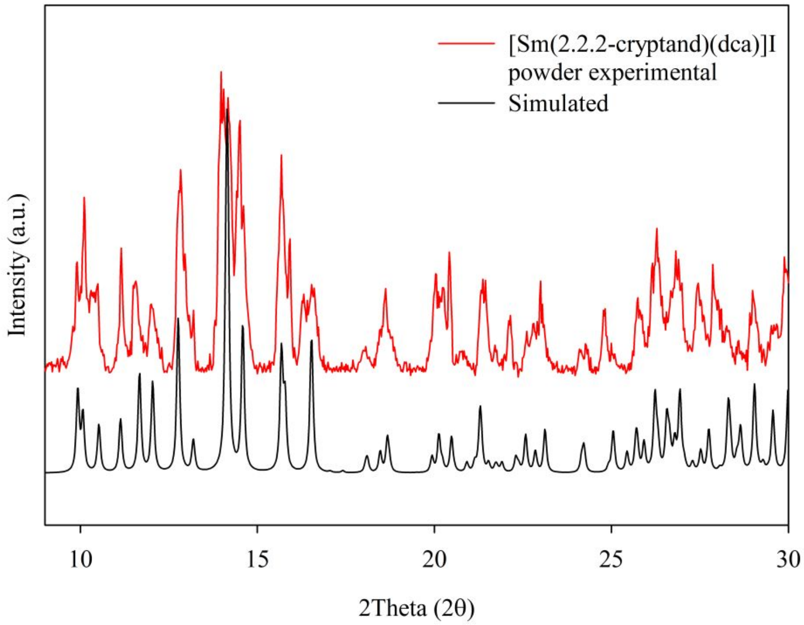

Figure S5. Powder X-ray diffractogram of [Sm(2.2.2-cryptand)(dca)]I (red, top) compared with the calculated powder diffractogram from the single crystal X-ray diffraction measurement of [Sm(2.2.2-cryptand)(dca)]I (black, bottom). Note that diffractograms are stacked.

UV-vis-NIR Spectroscopy

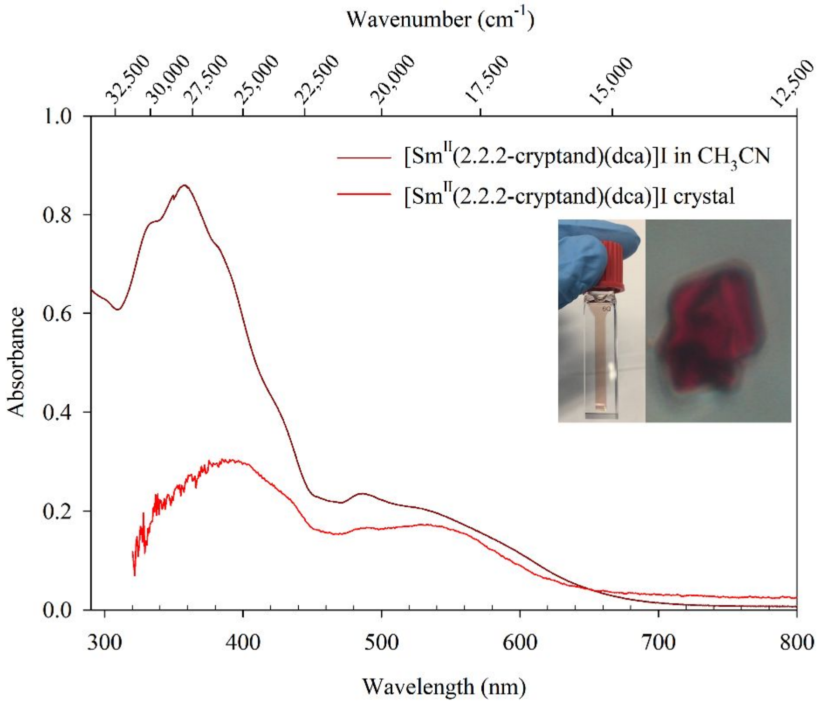

Figure S6. Comparison of the UV-vis-NIR spectra of solid-state [Sm(2.2.2-cryptand)(dca)]I (red) and an acetonitrile solution of the same complex (dark red; 0.97 mM), with associated images (not to scale). Note that an excellent match between the solid-state and solution phase UV-vis-NIR is rare for samarium(II) compounds, and such agreement suggests similar if not identical coordination environments of samarium(II) in the solid-state and solution phase.

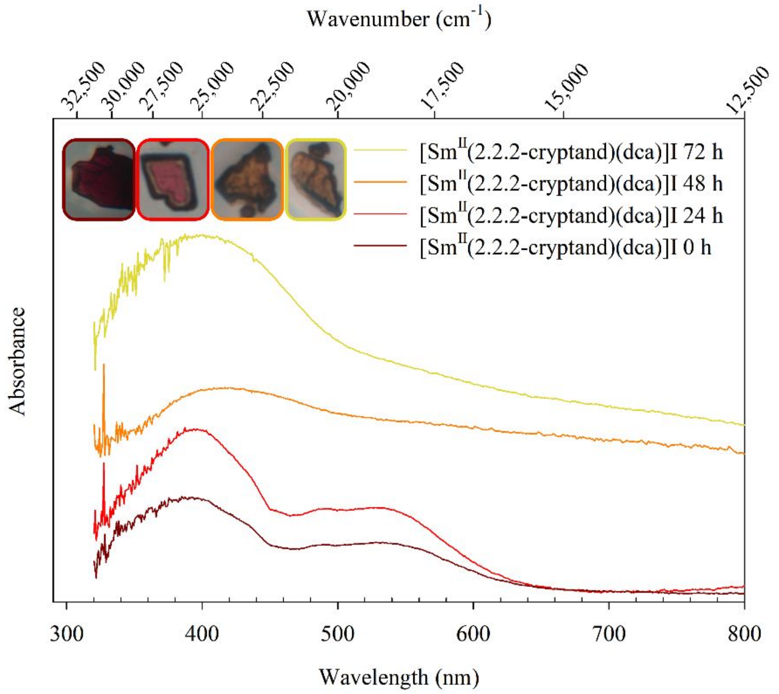

Figure S7. Solid-state UV-vis-NIR spectra of the degradation of single crystals of [Sm(2.2.2-cryptand)(dca)]I in immersion oil under normal atmospheric conditions over a period of several days. This experiment was used as a control for comparison with the high-pressure data. Note that the crystal images are not to scale to one another, and that spectra are stacked.

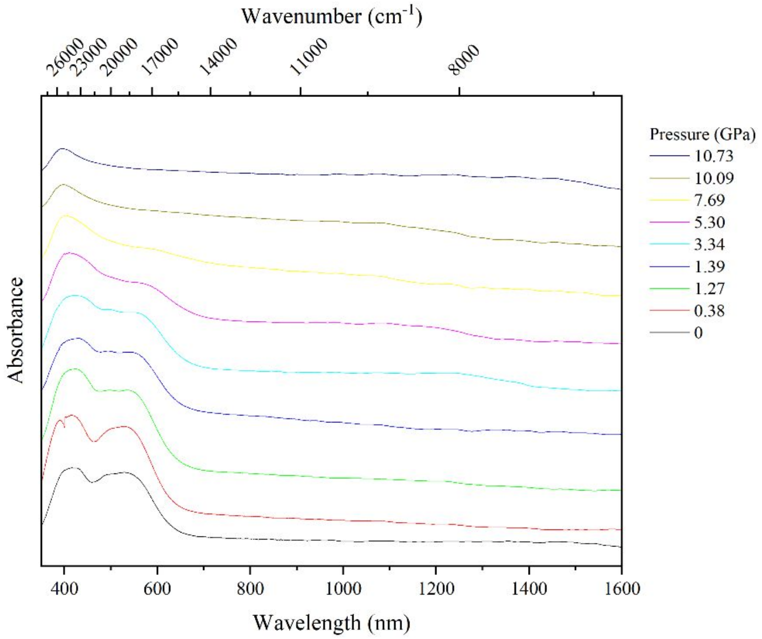

Figure S8. Solid-state UV-vis-NIR as a function of increasing pressure. A single crystal of [Sm(2.2.2-cryptand)(dca)]I was placed in a diamond anvil cell along with a ruby barometer to measure the pressure. Note that spectra are stacked.

Raman Spectroscopy

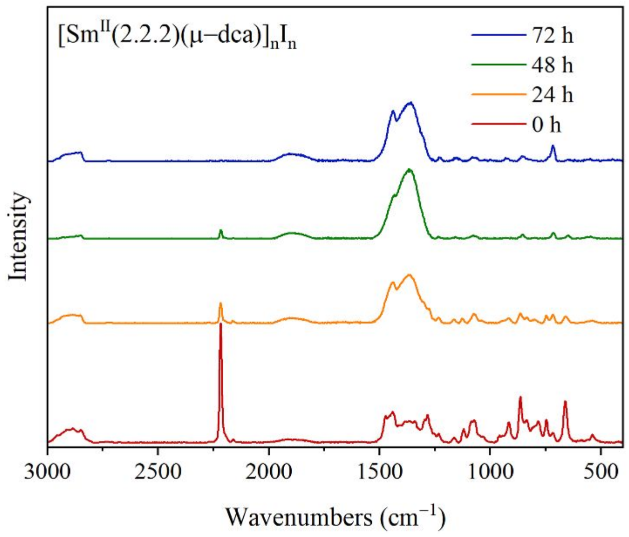

Figure S9. Raman spectra of the degradation of single crystals of [Sm(2.2.2-cryptand)(dca)]I in immersion oil under normal atmospheric conditions over a period of several days. This experiment was used as a control for comparison with the high-pressure data. Note that the spectra are stacked.

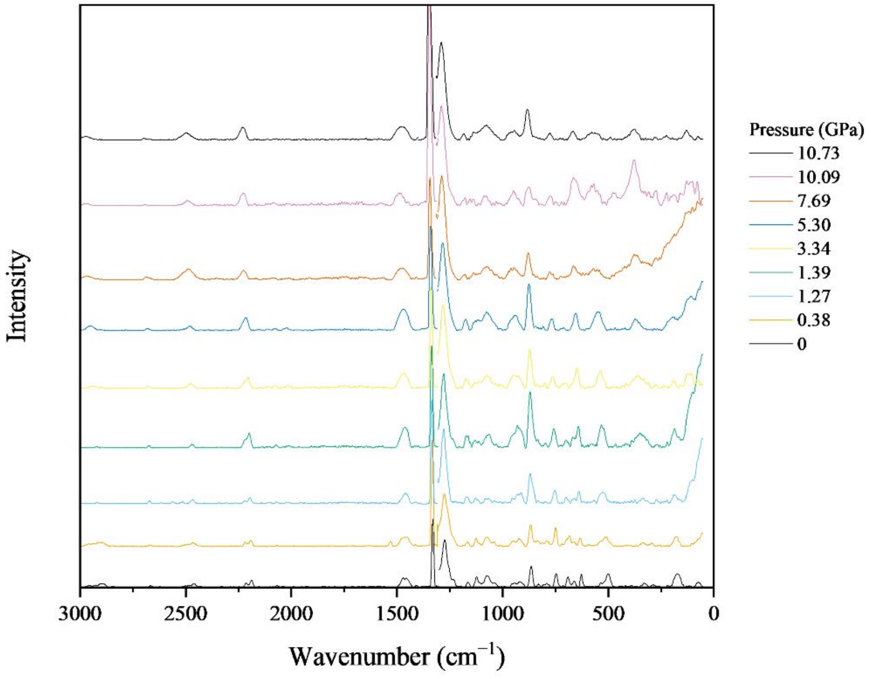

Figure S10. Solid-state Raman as a function of increasing pressure. A single crystal of [Sm(2.2.2-cryptand)(dca)]I was placed in a diamond anvil cell along with a ruby barometer. Note that spectra are stacked.

Table S8. Raman assignments for the high-pressure experiments on [Sm(2.2.2-cryptand)(dca)]I.

**Peak Energy (cm^{-1})**[a]	**Assignment**	**Reference**
373	Sm – N (ν_s)[b]	10
562	C – C – O deformation and C – O – C (δ)	11
777<br>795	N = C = N (δ)	10, 12, 13, 14
884<br>945	Mixture of C – O – C (ν_s) and CH_2 rocking	11
1139	C – N	12
1290	N = C = N (ν_s)	13, 14
1350	diamond	
1469	CH_2	
2230	C ≡ N (ν_s)	12, 15
2978	C – H (saturated)	

[a]Note that peak energies shift with increasing pressure, and the values shown represent the energy at a pressure of 5-8 GPa; [b]oxidation state of samarium is unclear.

Diamond Anvil Cell Control Experiment

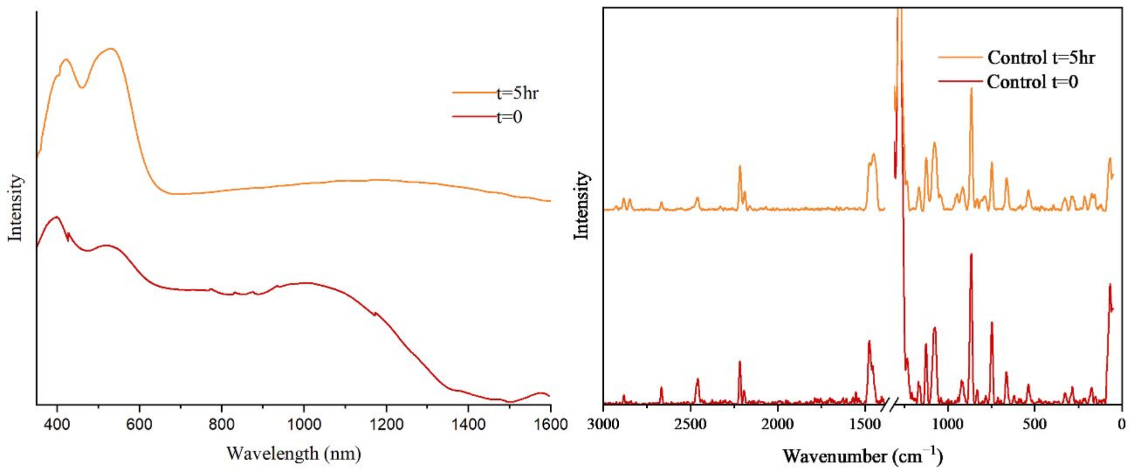

Figure S11. UV-vis-NIR and Raman spectra on a single crystal of [Sm(2.2.2-cryptand)(dca)]I at < 1 GPa of pressure before and after standing at otherwise ambient conditions for 5 h.

^{1}H-NMR Spectroscopy

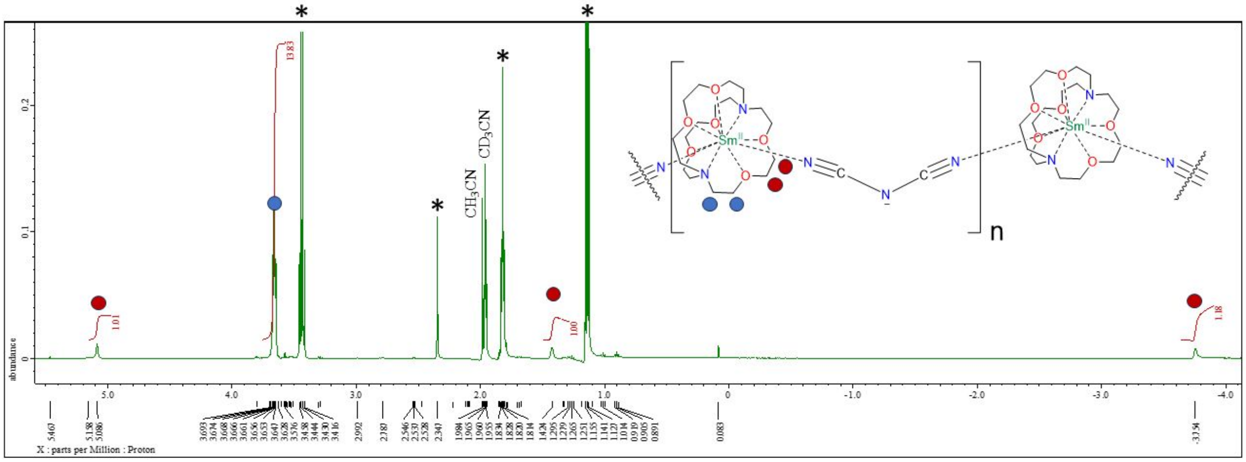

Figure S12. ^{1}H-NMR of [Sm(2.2.2-cryptand)(dca)]I in CD_3CN. Glovebox impurity solvents are indicated by * (and include diethyl ether, tetrahydrofuran, and toluene).

Computational Details

The optimization of different spin states for samarium dimers are computed by employing DFT hybrid functional[16] (B3PW91) along with small core pseudopotential Stuttgart basis set for samarium, iodine atoms[17] and Pople basis set[18] (6-31G**) for the rest of the atoms. Frequency calculations were performed to confirm minima and transition states for rest of the structures and for obtaining thermal corrections over the energies. All the calculations were performed using Gaussian 09 suite of programs.[19]

Table S9. Energetics for different spin states computed for [Sm(2.2.2-cryptand)(dca)]I

	ΔH (kcal/mol)	ΔG (kcal/mol)
s=6	2.9	0.0
s=0, open shell singlet	0.0	0.1

Table S10. Computed spin density for metal atoms in [Sm(2.2.2-cryptand)(dca)]I, s=6

Atom labels	Spin densities	
	s=6	s=0, open shell singlet
Sm1	6.024163	6.023808
Sm71	6.024674	-6.025032

Table S11. Computed natural charges for selected atoms in [Sm(2.2.2-cryptand)(dca)]I, s=6

Atom labels	Natural charges
Sm1	1.31574
O2	-0.58821
O3	-0.58474
O4	-0.60358
O5	-0.61309
O6	-0.58556
O7	-0.59160
N9	-0.54056
N10	-0.54778
N69	-0.60948
I70	-0.93548
Sm71	1.33053
O72	-0.59294
O73	-0.60697
O74	-0.59862

O75	-0.59973	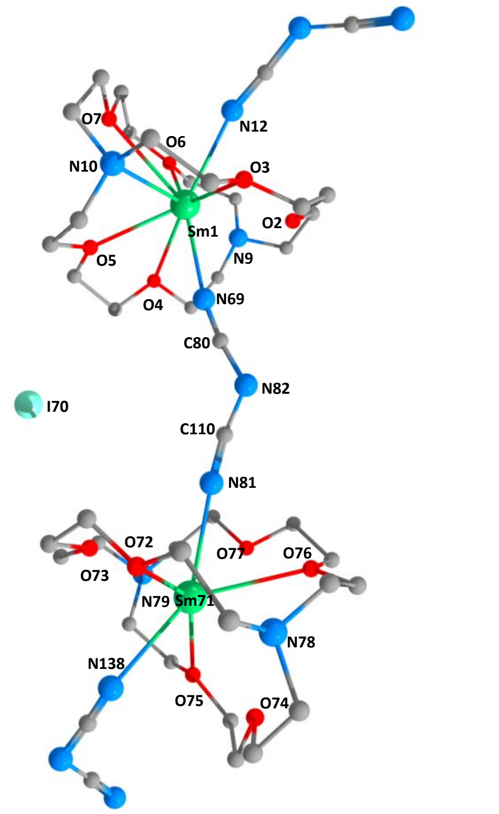
O76	-0.58416	
O77	-0.59184	
N78	-0.54339	
N79	-0.55365	
C80	0.55551	
N81	-0.60140	
N82	-0.68473	
C110	0.54186	
N138	-0.66693	

Table S12. Computed Wiberg bond index between selected atoms in [Sm(2.2.2-cryptand)(dca)]I, s=6

Atom labels	Wiberg bond index
Sm1-O2	0.0941
Sm1-O3	0.0994
Sm1-O4	0.0950
Sm1-O5	0.1168
Sm1-O6	0.0957
Sm1-O7	0.0911
Sm1-N9	0.0735
Sm1-N10	0.0887
Sm1-N12	0.2003
Sm1-N69	0.1844
N69-C80	2.5277
C80-N82	1.3299
N82-C110	1.3266
C110-N81	2.5351
Sm71-N81	0.1749
Sm71-O72	0.1036
Sm71-O73	0.1229
Sm71-O74	0.0872

Sm71-O75	0.1026
Sm71-O76	0.0855
Sm71-O77	0.0830
Sm71-N78	0.0746
Sm71-N79	0.0879
Sm71-N138	0.1848

Table S13. Bonding orbitals (Alpha molecular orbitals, AMO) of atoms in dicynamide linker in between metal atoms for [Sm(2.2.2-cryptand)(dca)]I

(0.99576) BD (1) N 69- C 80
(58.36%) 0.7639* N 69 s(46.32%)p 1.15(53.47%)d 0.00(0.22%)
(41.64%) 0.6453* C 80 s(49.86%)p 1.00(50.07%)d 0.00(0.07%)
(0.99120) BD (2) N 69- C 80
(59.53%) 0.7716* N 69 s(2.13%)p45.93(97.63%)d 0.12(0.25%)
(40.47%) 0.6362* C 80 s(1.10%)p89.42(98.76%)d 0.13(0.14%)
(0.98790) BD (3) N 69- C 80
(61.85%) 0.7864* N 69 s(0.01%)p99.99(99.75%)d19.88(0.24%)
(38.15%) 0.6177* C 80 s(0.01%)p 1.00(99.83%)d 0.00(0.16%)
(0.98904) BD (1) C 80- N 82
(39.95%) 0.6321* C 80 s(48.85%)p 1.05(51.06%)d 0.00(0.09%)
(60.05%) 0.7749* N 82 s(36.14%)p 1.76(63.74%)d 0.00(0.12%)
(0.99592) BD (1) N 81- C110
(58.17%) 0.7627* N 81 s(45.99%)p 1.17(53.80%)d 0.00(0.21%)
(41.83%) 0.6468* C110 s(49.87%)p 1.00(50.06%)d 0.00(0.07%)
(0.99138) BD (2) N 81- C110
(59.27%) 0.7698* N 81 s(2.21%)p44.05(97.53%)d 0.12(0.26%)
(40.73%) 0.6382* C110 s(1.11%)p88.65(98.74%)d 0.13(0.14%)
(0.98813) BD (3) N 81- C110
(61.68%) 0.7854* N 81 s(0.00%)p 1.00(99.76%)d 0.00(0.24%)
(38.32%) 0.6190* C110 s(0.00%)p 1.00(99.84%)d 0.00(0.16%)
(0.98937) BD (1) N 82- C110
(59.95%) 0.7743* N 82 s(36.41%)p 1.74(63.48%)d 0.00(0.11%)
(40.05%) 0.6329* C110 s(48.82%)p 1.05(51.09%)d 0.00(0.09%)

Table S14. DFT computed NBO second order perturbation analysis for [Sm(2.2.2-cryptand)(dca)]I

Donor NBO	Acceptor NBO	E(2) kcal/mol
(0.95569) LP (2) O 2<br>s(13.28%)p 6.53(86.67%)d 0.00(0.05%)	(0.04937) LV (4)Sm 1<br>s(0.01%)p<br>1.00(0.09%)d99.99(99.66%)f<br>1.61(0.14%)g 1.26(0.11%)	5.00
(0.95523) LP (2) O 3<br>s(13.25%)p 6.54(86.70%)d 0.00(0.05%)	(0.04937) LV (4)Sm 1	3.19

	s(0.01%)p 1.00(0.09%)d99.99(99.66%)f 1.61(0.14%)g 1.26(0.11%)	
(0.95523) LP (2) O 3 s(13.25%)p 6.54(86.70%)d 0.00(0.05%)	(0.04089) LV (5)Sm 1 s(0.40%)p 0.79(0.32%)d99.99(98.55%)f 1.26(0.50%)g 0.58(0.23%)	3.91
(0.95678) LP (2) O 4 s(14.31%)p 5.99(85.64%)d 0.00(0.05%)	(0.04937) LV (4)Sm 1 s(0.01%)p 1.00(0.09%)d99.99(99.66%)f 1.61(0.14%)g 1.26(0.11%)	3.44
(0.96194) LP (2) O 5 s(35.27%)p 1.83(64.70%)d 0.00(0.03%)	(0.06393) LV (3)Sm 1 s(0.39%)p 0.07(0.03%)d99.99(99.23%)f 0.84(0.33%)g 0.05(0.02%)	6.76
(0.96194) LP (2) O 5 s(35.27%)p 1.83(64.70%)d 0.00(0.03%)	(0.04937) LV (4)Sm 1 s(0.01%)p 1.00(0.09%)d99.99(99.66%)f 1.61(0.14%)g 1.26(0.11%)	3.49
(0.96513) LP (1) O 6 s(25.58%)p 2.91(74.36%)d 0.00(0.06%)	(0.06393) LV (3)Sm 1 s(0.39%)p 0.07(0.03%)d99.99(99.23%)f 0.84(0.33%)g 0.05(0.02%)	3.05
(0.95580) LP (2) O 7 s(12.47%)p 7.01(87.47%)d 0.00(0.06%)	(0.03461) LV (6)Sm 1 s(0.12%)p 0.25(0.03%)d99.99(99.32%)f 2.96(0.35%)g 1.55(0.18%)	2.71
(0.95580) LP (2) O 7 s(12.47%)p 7.01(87.47%)d 0.00(0.06%)	(0.06393) LV (3)Sm 1 s(0.39%)p 0.07(0.03%)d99.99(99.23%)f 0.84(0.33%)g 0.05(0.02%)	2.62
(0.93326) LP (1) N 9 s(15.88%)p 5.30(84.08%)d 0.00(0.05%)	(0.06636) LV (2)Sm 1 s(19.60%)p 0.00(0.04%)d 4.07(79.86%)f 0.02(0.44%)g 0.00(0.06%)	4.55
(0.93326) LP (1) N 9 s(15.88%)p 5.30(84.08%)d 0.00(0.05%)	(0.04089) LV (5)Sm 1 s(0.40%)p 0.79(0.32%)d99.99(98.55%)f 1.26(0.50%)g 0.58(0.23%)	2.78
(0.93071) LP (1) N 10 s(15.68%)p 5.38(84.29%)d 0.00(0.04%)	(0.06636) LV (2)Sm 1 s(19.60%)p 0.00(0.04%)d 4.07(79.86%)f 0.02(0.44%)g 0.00(0.06%)	5.76
(0.93071) LP (1) N 10 s(15.68%)p 5.38(84.29%)d 0.00(0.04%)	(0.04089) LV (5)Sm 1	3.83

	s(0.40%)p<br>0.79(0.32%)d99.99(98.55%)f<br>1.26(0.50%)g 0.58(0.23%)	
(0.93931) LP (1) N 12<br>s(51.02%)p 0.96(48.95%)d 0.00(0.03%)	(0.08732) LV (1)Sm 1<br>s(79.43%)p 0.00(0.02%)d<br>0.26(20.27%)f 0.00(0.27%)g<br>0.00(0.02%)	18.58
(0.93931) LP (1) N 12<br>s(51.02%)p 0.96(48.95%)d 0.00(0.03%)	(0.06636) LV (2)Sm 1<br>s(19.60%)p 0.00(0.04%)d<br>4.07(79.86%)f 0.02(0.44%)g<br>0.00(0.06%)	11.12
(0.93931) LP (1) N 12<br>s(51.02%)p 0.96(48.95%)d 0.00(0.03%)	(0.06393) LV (3)Sm 1<br>s(0.39%)p<br>0.07(0.03%)d99.99(99.23%)f<br>0.84(0.33%)g 0.05(0.02%)	4.98
(0.94145) LP (1) N 69<br>s(51.57%)p 0.94(48.39%)d 0.00(0.03%)	(0.08732) LV (1)Sm 1<br>s(79.43%)p 0.00(0.02%)d<br>0.26(20.27%)f 0.00(0.27%)g<br>0.00(0.02%)	16.41
(0.94145) LP (1) N 69<br>s(51.57%)p 0.94(48.39%)d 0.00(0.03%)	(0.06636) LV (2)Sm 1<br>s(19.60%)p 0.00(0.04%)d<br>4.07(79.86%)f 0.02(0.44%)g<br>0.00(0.06%)	4.25
(0.94145) LP (1) N 69<br>s(51.57%)p 0.94(48.39%)d 0.00(0.03%)	(0.06393) LV (3)Sm 1<br>s(0.39%)p<br>0.07(0.03%)d99.99(99.23%)f<br>0.84(0.33%)g 0.05(0.02%)	13.24
(0.94335) LP (1) N 81<br>s(51.77%)p 0.93(48.20%)d 0.00(0.03%)	(0.08578) LV (1)Sm 71<br>s(81.07%)p 0.00(0.12%)d<br>0.23(18.69%)f 0.00(0.10%)g<br>0.00(0.02%)	15.88
(0.94335) LP (1) N 81<br>s(51.77%)p 0.93(48.20%)d 0.00(0.03%)	(0.06724) LV (2)Sm 71<br>s(18.09%)p 0.00(0.04%)d<br>4.50(81.42%)f 0.02(0.40%)g<br>0.00(0.05%)	5.87
(0.94335) LP (1) N 81<br>s(51.77%)p 0.93(48.20%)d 0.00(0.03%)	(0.05939) LV (3)Sm 71<br>s(0.21%)p<br>0.14(0.03%)d99.99(99.46%)f<br>1.39(0.29%)g 0.10(0.02%)	9.26
(0.96989) LP (1) O 72<br>s(27.99%)p 2.57(71.94%)d 0.00(0.07%)	(0.05050) LV (4)Sm 71<br>s(0.04%)p<br>3.59(0.13%)d99.99(99.41%)f<br>9.24(0.33%)g 2.73(0.10%)	3.44
(0.95495) LP (2) O 72<br>s(14.23%)p 6.02(85.73%)d 0.00(0.04%)	(0.05050) LV (4)Sm 71	4.44

	s(0.04%)p<br>3.59(0.13%)d99.99(99.41%)f<br>9.24(0.33%)g 2.73(0.10%)	
(0.95495) LP (2) O 72<br>s(14.23%)p 6.02(85.73%)d 0.00(0.04%)	(0.04003) LV (5)Sm 71<br>s(0.34%)p<br>1.08(0.37%)d99.99(98.71%)f<br>0.98(0.34%)g 0.69(0.24%)	3.17
(0.96696) LP (1) O 73<br>s(22.30%)p 3.48(77.64%)d 0.00(0.06%)	(0.05050) LV (4)Sm 71<br>s(0.04%)p<br>3.59(0.13%)d99.99(99.41%)f<br>9.24(0.33%)g 2.73(0.10%)	4.23
(0.95772) LP (2) O 73<br>s(19.47%)p 4.14(80.51%)d 0.00(0.03%)	(0.05050) LV (4)Sm 71<br>s(0.04%)p<br>3.59(0.13%)d99.99(99.41%)f<br>9.24(0.33%)g 2.73(0.10%)	6.76
(0.95735) LP (2) O 74<br>s(12.04%)p 7.30(87.90%)d 0.00(0.06%)	(0.05050) LV (4)Sm 71<br>s(0.04%)p<br>3.59(0.13%)d99.99(99.41%)f<br>9.24(0.33%)g 2.73(0.10%)	3.11
(0.96501) LP (1) O 75<br>s(30.04%)p 2.33(69.91%)d 0.00(0.05%)	(0.05939) LV (3)Sm 71<br>s(0.21%)p<br>0.14(0.03%)d99.99(99.46%)f<br>1.39(0.29%)g 0.10(0.02%)	5.70
(0.96139) LP (2) O 75<br>s(11.82%)p 7.46(88.14%)d 0.00(0.05%)	(0.05939) LV (3)Sm 71<br>s(0.21%)p<br>0.14(0.03%)d99.99(99.46%)f<br>1.39(0.29%)g 0.10(0.02%)	3.21
(0.96695) LP (1) O 76<br>s(31.10%)p 2.21(68.83%)d 0.00(0.07%)	(0.05939) LV (3)Sm 71<br>s(0.21%)p<br>0.14(0.03%)d99.99(99.46%)f<br>1.39(0.29%)g 0.10(0.02%)	2.74
(0.96695) LP (1) O 76<br>s(31.10%)p 2.21(68.83%)d 0.00(0.07%)	(0.03143) LV (6)Sm 71<br>s(0.07%)p<br>0.70(0.05%)d99.99(99.41%)f<br>4.02(0.27%)g 3.20(0.21%)	2.55
(0.93367) LP (1) N 78<br>s(16.01%)p 5.24(83.94%)d 0.00(0.05%)	(0.06724) LV (2)Sm 71<br>s(18.09%)p 0.00(0.04%)d<br>4.50(81.42%)f 0.02(0.40%)g<br>0.00(0.05%)	4.38
(0.93367) LP (1) N 78<br>s(16.01%)p 5.24(83.94%)d 0.00(0.05%)	(0.04003) LV (5)Sm 71<br>s(0.34%)p<br>1.08(0.37%)d99.99(98.71%)f<br>0.98(0.34%)g 0.69(0.24%)	3.12
(0.93242) LP (1) N 79<br>s(16.29%)p 5.14(83.68%)d 0.00(0.04%)	(0.06724) LV (2)Sm 71	5.02

	s(18.09%)p 0.00(0.04%)d 4.50(81.42%)f 0.02(0.40%)g 0.00(0.05%)	
(0.93242) LP (1) N 79 s(16.29%)p 5.14(83.68%)d 0.00(0.04%)	(0.04003) LV (5)Sm 71 s(0.34%)p 1.08(0.37%)d99.99(98.71%)f 0.98(0.34%)g 0.69(0.24%)	3.60
(0.94348) LP (1) N138 s(52.37%)p 0.91(47.57%)d 0.00(0.06%)	(0.08578) LV (1)Sm 71 s(81.07%)p 0.00(0.12%)d 0.23(18.69%)f 0.00(0.10%)g 0.00(0.02%)	14.24
(0.94348) LP (1) N138 s(52.37%)p 0.91(47.57%)d 0.00(0.06%)	(0.06724) LV (2)Sm 71 s(18.09%)p 0.00(0.04%)d 4.50(81.42%)f 0.02(0.40%)g 0.00(0.05%)	8.08
(0.94348) LP (1) N138 s(52.37%)p 0.91(47.57%)d 0.00(0.06%)	(0.05939) LV (3)Sm 71 s(0.21%)p 0.14(0.03%)d99.99(99.46%)f 1.39(0.29%)g 0.10(0.02%)	6.25

Table S15. Computed MOs (AMO) for [Sm(2.2.2-cryptand)(dca)]I, s=6. (a)HOMO-22 (b)HOMO-19 (c)HOMO-12 (d)HOMO-11 (e)HOMO-10 (f)HOMO-9 (g)HOMO-8 (h)HOMO-7 (i)HOMO-6 (j)HOMO-5 (k)HOMO-4 (l)HOMO-3 (m)HOMO-2 (n)HOMO-1 (o)HOMO (p)LUMO

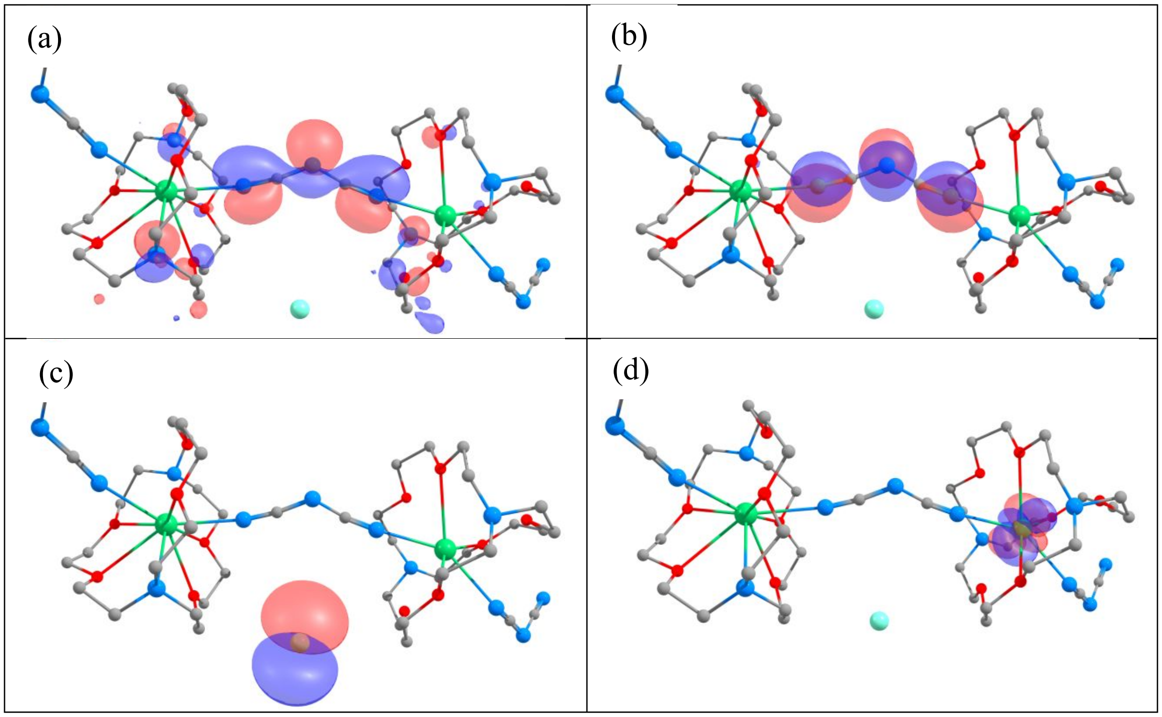

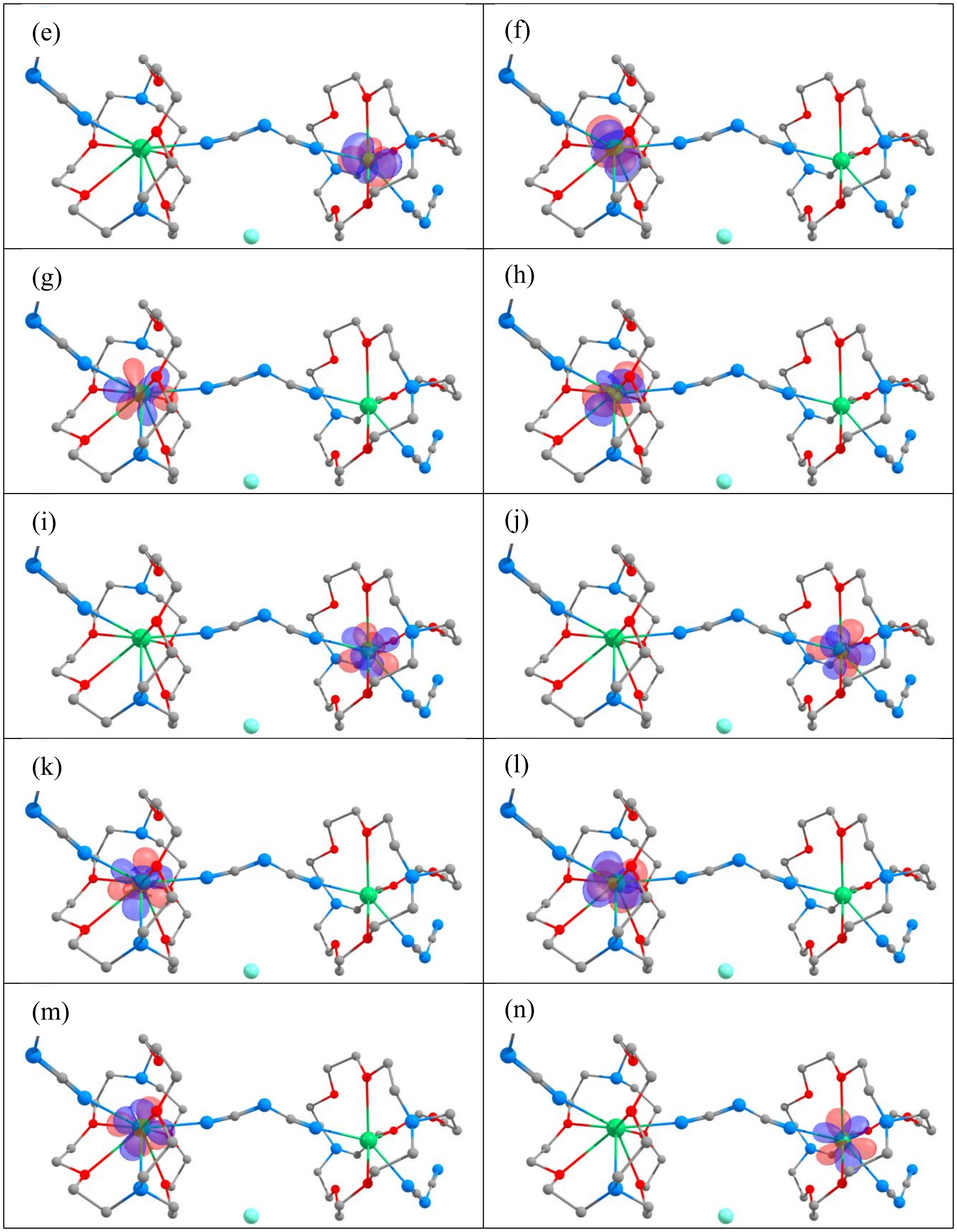

(e)
(f)
(g)
(h)
(i)
(j)
(k)
(l)
(m)
(n)

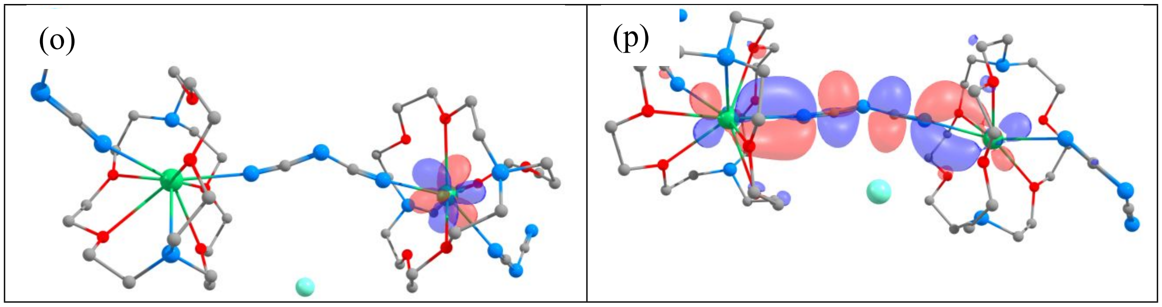

Table S16. Computed natural charges for selected atoms in 2, s=5.

Atom labels	Natural charges
Sm1	1.60920
O2	-0.58230
O3	-0.58152
O4	-0.60866
O5	-0.61732
O6	-0.58399
O7	-0.58560
N9	-0.54241
N10	-0.54631
N69	-0.78299
I70	-0.92753
Sm71	1.60546
O72	-0.59924
O73	-0.61471
O74	-0.58964
O75	-0.60170
O76	-0.59969
O77	-0.58827
N78	-0.55133
N79	-0.54618
C80	0.38702
N81	-0.80025
N82	-0.55782
C110	0.03697
N138	-0.69062

Table S17. Computed Wiberg bond index between selected atoms in **2**, s=5

Atom labels	Wiberg bond index
Sm1-O2	0.1203
Sm1-O3	0.1220
Sm1-O4	0.1249
Sm1-O5	0.1565
Sm1-O6	0.1180

Sm1-O7	0.1152
Sm1-N9	0.1057
Sm1-N10	0.1366
Sm1-N12	0.2633
Sm1-N69	0.4643
N69-C80	1.7697
C80-N82	2.1696
N82-C110	0.0006
C110-N81	2.6381
Sm71-N81	0.2856
Sm71-O72	0.1488
Sm71-O73	0.1654
Sm71-O74	0.1212
Sm71-O75	0.1399
Sm71-O76	0.1236
Sm71-O77	0.0986
Sm71-N78	0.1288
Sm71-N79	0.1163
Sm71-N138	0.2460

Table S18. Computed spin density for metal atoms in 2, s=5

Atom labels	Spin densities
	s=5
Sm1	5.450884
Sm71	5.458973
N69	-0.514405
C80	0.195070
N81	-0.015180
N82	-0.490638
C110	0.003057

Bonding orbitals (Alpha molecular orbitals, AMO) between selected atoms in 2, s=5.
(0.99373) BD (1) N 69- C 80
(57.70%) 0.7596* N 69 s(46.23%)p 1.16(53.61%)d 0.00(0.16%)
(42.30%) 0.6504* C 80 s(49.28%)p 1.03(50.63%)d 0.00(0.08%)
(0.79700) BD (2) N 69- C 80
(38.53%) 0.6207* N 69 s(0.00%)p 1.00(99.82%)d 0.00(0.18%)
(61.47%) 0.7840* C 80 s(0.00%)p 1.00(99.99%)d 0.00(0.00%)
(0.99584) BD (1) C 80- N 82
(43.75%) 0.6614* C 80 s(50.49%)p 0.98(49.44%)d 0.00(0.06%)
(56.25%) 0.7500* N 82 s(41.98%)p 1.38(57.75%)d 0.01(0.27%)
(0.98855) BD (2) N 81- C110
(67.21%) 0.8198* N 81 s(0.02%)p99.99(99.80%)d 7.04(0.17%)
(32.79%) 0.5726* C110 s(0.01%)p 1.00(99.66%)d 0.00(0.33%)

(0.98433) BD (3) N 81- C110
(67.43%) 0.8212* N 81 s(0.02%)p99.99(99.81%)d 8.98(0.17%)
(32.57%) 0.5707* C110 s(0.01%)p 1.00(99.67%)d 0.00(0.32%)

Table S19. DFT computed NBO second order perturbation analysis (AMO) for 2

Donor NBO	Acceptor NBO	E(2) kcal/mol
(0.95105) LP (2) O 2<br>s(22.02%)p 3.54(77.94%)d 0.00(0.04%)	(0.07151) LV (4)Sm 1<br>s(0.68%)p<br>0.13(0.09%)d99.99(98.59%)f<br>0.83(0.57%)g 0.09(0.06%)	4.69
(0.95105) LP (2) O 2<br>s(22.02%)p 3.54(77.94%)d 0.00(0.04%)	(0.06622) LV (5)Sm 1<br>s(1.85%)p<br>0.03(0.06%)d51.99(96.31%)f<br>0.92(1.71%)g 0.04(0.07%)	4.67
(0.94990) LP (2) O 3<br>s(19.72%)p 4.07(80.24%)d 0.00(0.04%)	(0.07151) LV (4)Sm 1<br>s(0.68%)p<br>0.13(0.09%)d99.99(98.59%)f<br>0.83(0.57%)g 0.09(0.06%)	6.30
(0.94990) LP (2) O 3<br>s(19.72%)p 4.07(80.24%)d 0.00(0.04%)	(0.05824) LV (6)Sm 1<br>s(0.79%)p<br>0.09(0.07%)d99.99(97.73%)f<br>1.64(1.29%)g 0.16(0.13%)	5.18
(0.95154) LP (2) O 4<br>s(21.14%)p 3.73(78.82%)d 0.00(0.04%)	(0.09317) LV (2)Sm 1<br>s(33.87%)p 0.00(0.11%)d<br>1.92(64.91%) f 0.03(1.09%)g<br>0.00(0.03%)	6.47
(0.95154) LP (2) O 4<br>s(21.14%)p 3.73(78.82%)d 0.00(0.04%)	(0.07151) LV (4)Sm 1<br>s(0.68%)p<br>0.13(0.09%)d99.99(98.59%)f<br>0.83(0.57%)g 0.09(0.06%)	4.45
(0.95256) LP (2) O 5<br>s(31.30%)p 2.19(68.69%)d 0.00(0.01%)	(0.09317) LV (2)Sm 1<br>s(33.87%)p 0.00(0.11%)d<br>1.92(64.91%) f 0.03(1.09%)g<br>0.00(0.03%)	11.14
(0.95256) LP (2) O 5<br>s(31.30%)p 2.19(68.69%)d 0.00(0.01%)	(0.06622) LV (5)Sm 1<br>s(1.85%)p<br>0.03(0.06%)d51.99(96.31%)f<br>0.92(1.71%)g 0.04(0.07%)	10.90
(0.95462) LP (2) O 6<br>s(25.25%)p 2.96(74.71%)d 0.00(0.04%)	(0.08692) LV (3)Sm 1<br>s(46.61%)p 0.00(0.10%)d<br>1.13(52.50%)f 0.02(0.75%)g<br>0.00(0.04%)	4.99
(0.95462) LP (2) O 6<br>s(25.25%)p 2.96(74.71%)d 0.00(0.04%)	(0.07151) LV (4)Sm 1	3.90

	s(0.68%)p<br>0.13(0.09%)d99.99(98.59%)f<br>0.83(0.57%)g 0.09(0.06%)	
(0.95118) LP (2) O 7<br>s(17.72%)p 4.64(82.23%)d 0.00(0.04%)	(0.08692) LV (3)Sm 1<br>s(46.61%)p 0.00(0.10%)d<br>1.13(52.50%)f 0.02(0.75%)g<br>0.00(0.04%)	3.69
(0.95118) LP (2) O 7<br>s(17.72%)p 4.64(82.23%)d 0.00(0.04%)	(0.07151) LV (4)Sm 1<br>s(0.68%)p<br>0.13(0.09%)d99.99(98.59%)f<br>0.83(0.57%)g 0.09(0.06%)	5.85
(0.92745) LP (1) N 9<br>s(16.33%)p 5.12(83.63%)d 0.00(0.04%)	(0.08692) LV (3)Sm 1<br>s(46.61%)p 0.00(0.10%)d<br>1.13(52.50%)f 0.02(0.75%)g<br>0.00(0.04%)	5.57
(0.92043) LP (1) N 10<br>s(16.23%)p 5.16(83.74%)d 0.00(0.03%	(0.08692) LV (3)Sm 1<br>s(46.61%)p 0.00(0.10%)d<br>1.13(52.50%)f 0.02(0.75%)g<br>0.00(0.04%)	7.30
(0.92043) LP (1) N 10<br>s(16.23%)p 5.16(83.74%)d 0.00(0.03%	(0.05824) LV (6)Sm 1<br>s(0.79%)p<br>0.09(0.07%)d99.99(97.73%)f<br>1.64(1.29%)g 0.16(0.13%)	3.91
(0.92706) LP (1) N 12<br>s(49.49%)p 1.02(50.49%)d 0.00(0.02%)	(0.10904) LV (1)Sm 1<br>s(16.09%)p 0.01(0.12%)d<br>5.16(83.07%)f 0.04(0.68%)g<br>0.00(0.04%)	20.00
(0.92706) LP (1) N 12<br>s(49.49%)p 1.02(50.49%)d 0.00(0.02%)	(0.09317) LV (2)Sm 1<br>s(33.87%)p 0.00(0.11%)d<br>1.92(64.91%) f 0.03(1.09%)g<br>0.00(0.03%)	17.35
(0.92706) LP (1) N 12<br>s(49.49%)p 1.02(50.49%)d 0.00(0.02%)	(0.08692) LV (3)Sm 1<br>s(46.61%)p 0.00(0.10%)d<br>1.13(52.50%)f 0.02(0.75%)g<br>0.00(0.04%)	3.56
(0.92332) LP (1) N 69<br>s(53.65%)p 0.86(46.32%)d 0.00(0.03%)	(0.10904) LV (1)Sm 1<br>s(16.09%)p 0.01(0.12%)d<br>5.16(83.07%)f 0.04(0.68%)g<br>0.00(0.04%)	39.47
(0.95125) LP (2) O 72<br>s(33.44%)p 1.99(66.54%)d 0.00(0.02%)	(0.08898) LV (2)Sm 71<br>s(64.99%)p 0.00(0.08%)d<br>0.54(34.83%)f 0.00(0.07%)g<br>0.00(0.04%)	4.40
(0.95125) LP (2) O 72<br>s(33.44%)p 1.99(66.54%)d 0.00(0.02%)	(0.07290) LV (4)Sm 71	5.32

	s(0.50%)p 0.11(0.06%)d99.99(99.18%)f 0.37(0.18%)g 0.16(0.08%)	
(0.95125) LP (2) O 72 s(33.44%)p 1.99(66.54%)d 0.00(0.02%)	(0.06411) LV (5)Sm 71 s(1.19%)p 0.15(0.18%)d82.16(98.11%)f 0.32(0.39%)g 0.10(0.12%)	9.89
(0.95372) LP (2) O 73 s(40.20%)p 1.49(59.78%)d 0.00(0.01%)	(0.08898) LV (2)Sm 71 s(64.99%)p 0.00(0.08%)d 0.54(34.83%)f 0.00(0.07%)g 0.00(0.04%)	5.58
(0.95372) LP (2) O 73 s(40.20%)p 1.49(59.78%)d 0.00(0.01%)	(0.07290) LV (4)Sm 71 s(0.50%)p 0.11(0.06%)d99.99(99.18%)f 0.37(0.18%)g 0.16(0.08%)	17.21
(0.95126) LP (2) O 74 s(18.23%)p 4.48(81.73%)d 0.00(0.04%)	(0.07290) LV (4)Sm 71 s(0.50%)p 0.11(0.06%)d99.99(99.18%)f 0.37(0.18%)g 0.16(0.08%)	4.50
(0.95650) LP (2) O 75 s(36.62%)p 1.73(63.35%)d 0.00(0.02%)	(0.08413) LV (3)Sm 71 s(0.53%)p 0.29(0.15%)d99.99(98.83%)f 0.89(0.47%)g 0.04(0.02%)	6.41
(0.95650) LP (2) O 75 s(36.62%)p 1.73(63.35%)d 0.00(0.02%)	(0.05088) LV (6)Sm 71 s(0.40%)p 0.18(0.07%)d99.99(99.08%)f 0.84(0.34%)g 0.26(0.11%)	4.15
(0.95461) LP (2) O 76 s(28.34%)p 2.53(71.63%)d 0.00(0.03%)	(0.08413) LV (3)Sm 71 s(0.53%)p 0.29(0.15%)d99.99(98.83%)f 0.89(0.47%)g 0.04(0.02%)	6.08
(0.95461) LP (2) O 76 s(28.34%)p 2.53(71.63%)d 0.00(0.03%)	(0.07290) LV (4)Sm 71 s(0.50%)p 0.11(0.06%)d99.99(99.18%)f 0.37(0.18%)g 0.16(0.08%)	5.20
(0.95296) LP (2) O 77 s(14.65%)p 5.82(85.29%)d 0.00(0.06%)	(0.08413) LV (3)Sm 71 s(0.53%)p 0.29(0.15%)d99.99(98.83%)f 0.89(0.47%)g 0.04(0.02%)	4.17
(0.92113) LP (1) N 78 s(15.16%)p 5.59(84.81%)d 0.00(0.02%)	(0.08898) LV (2)Sm 71 s(64.99%)p 0.00(0.08%)d 0.54(34.83%)f 0.00(0.07%)g 0.00(0.04%)	8.96
(0.92113) LP (1) N 78 s(15.16%)p 5.59(84.81%)d 0.00(0.02%)	(0.06411) LV (5)Sm 71	4.73

	s(1.19%)p<br>0.15(0.18%)d82.16(98.11%)f<br>0.32(0.39%)g 0.10(0.12%)	
(0.92316) LP (1) N 79<br>s(15.78%)p 5.33(84.19%)d 0.00(0.03%)	(0.08898) LV (2)Sm 71<br>s(64.99%)p 0.00(0.08%)d<br>0.54(34.83%)f 0.00(0.07%)g<br>0.00(0.04%)	7.98
(0.92445) LP (1) N 81<br>s(43.93%)p 1.28(56.05%)d 0.00(0.02%)	(0.11772) LV (1)Sm 71<br>s(32.16%)p 0.00(0.09%)d<br>2.09(67.32%)f 0.01(0.41%)g<br>0.00(0.03%)	37.24
(0.93073) LP (1) N138<br>s(51.21%)p 0.95(48.74%)d 0.00(0.05%)	(0.11772) LV (1)Sm 71<br>s(32.16%)p 0.00(0.09%)d<br>2.09(67.32%)f 0.01(0.41%)g<br>0.00(0.03%)	21.75
(0.93073) LP (1) N138<br>s(51.21%)p 0.95(48.74%)d 0.00(0.05%)	(0.08413) LV (3)Sm 71<br>s(0.53%)p<br>0.29(0.15%)d99.99(98.83%)f<br>0.89(0.47%)g 0.04(0.02%)	13.28

Table S20. Computed MOs (AMO) for 2. (a)HOMO-40 (b)HOMO-37 (c)HOMO-33 (d)HOMO-30 (e)HOMO-29 (f)HOMO-21 (g)HOMO-19 (h)HOMO-16 (i)HOMO-14 (j)HOMO-13 (k)HOMO-12 (l)HOMO-8 (m)HOMO-4 (o)HOMO-1 (p)HOMO (q)LUMO

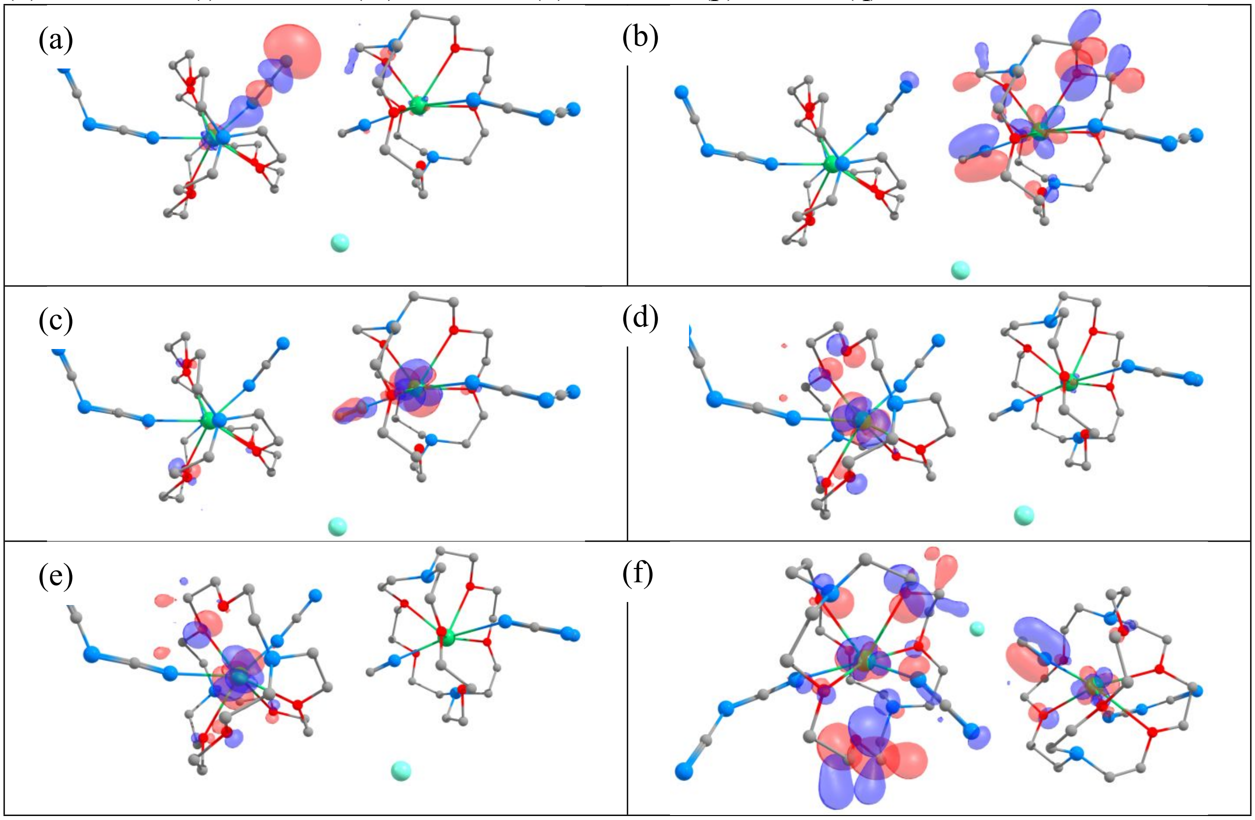

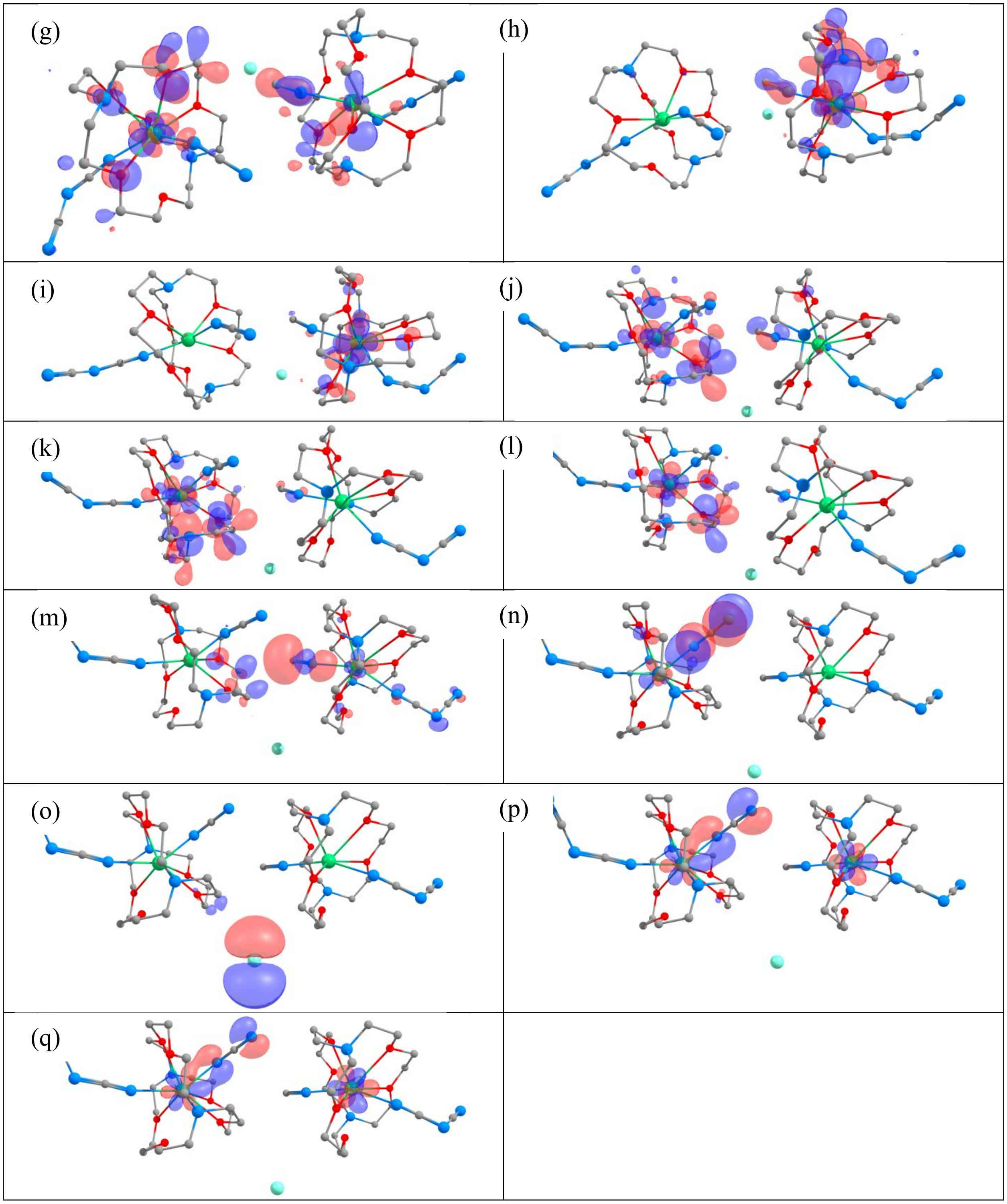

Optimized geometries

[Sm(2.2.2-cryptand)(dca)]I, s=6

Sm	8.056943000	5.667769000	8.398166000
O	8.103880000	6.014490000	11.206395000
O	5.687994000	5.630816000	9.864878000

O	10.384536000	4.142224000	7.839064000
O	8.022329000	3.864160000	6.415337000
O	9.909848000	7.472378000	7.329161000
O	7.495067000	7.098712000	6.000536000
N	5.412387000	10.258242000	12.238722000
N	10.733006000	6.195284000	9.807423000
N	5.553892000	5.131978000	6.958867000
C	5.961421000	10.182313000	11.204334000
N	7.419808000	8.114914000	8.972957000
N	6.582016000	10.178196000	10.037080000
C	9.167588000	6.819481000	11.678760000
H	9.149068000	6.857000000	12.779147000
H	9.054971000	7.848887000	11.311324000
C	4.425005000	5.372396000	7.867601000
H	3.498335000	4.918543000	7.470236000
H	4.258888000	6.452885000	7.923975000
C	10.474467000	6.180542000	11.253976000
H	11.303753000	6.660963000	11.805778000
H	10.434776000	5.134936000	11.575766000
C	6.860817000	6.354429000	11.802662000
H	6.600546000	7.398738000	11.592144000
H	6.921648000	6.228963000	12.894760000
C	9.248872000	3.338257000	5.909005000
H	9.732787000	4.094548000	5.274706000
H	9.066952000	2.427684000	5.326738000
C	5.645915000	3.705011000	6.603433000
H	4.745565000	3.365014000	6.059855000
H	5.704261000	3.131564000	7.532245000
C	11.742637000	5.174421000	9.478343000
H	12.413826000	4.988501000	10.332508000
H	12.374023000	5.537316000	8.663456000
C	5.798790000	5.431319000	11.264710000
H	6.052712000	4.384737000	11.479436000
H	4.843444000	5.672513000	11.755075000
C	6.863569000	3.348741000	5.773691000
H	6.929509000	2.253148000	5.706438000
H	6.798812000	3.744513000	4.748703000
C	6.996145000	9.075587000	9.520916000
C	8.245865000	8.290773000	5.850693000
H	7.948550000	9.022398000	6.612701000
H	8.078962000	8.720149000	4.849740000
C	6.093432000	7.330496000	5.928831000
H	5.862976000	7.954575000	5.051553000

H	5.764598000	7.867468000	6.829800000
C	11.200263000	7.694960000	7.857151000
H	11.521471000	8.726373000	7.645110000
H	11.931309000	7.023665000	7.382454000
C	11.120845000	3.872238000	9.019065000
H	11.923713000	3.149407000	8.807295000
H	10.461273000	3.433695000	9.781717000
C	10.123017000	2.964023000	7.074169000
H	9.608699000	2.206571000	7.677254000
H	11.068900000	2.538512000	6.706898000
C	11.153290000	7.529194000	9.366039000
H	12.145681000	7.797364000	9.775107000
H	10.433294000	8.258688000	9.746725000
C	4.634931000	4.884098000	9.286826000
H	3.702488000	5.056329000	9.846832000
H	4.858978000	3.809093000	9.336925000
C	5.409980000	5.988062000	5.770309000
H	4.348300000	6.142868000	5.516395000
H	5.869063000	5.492973000	4.912469000
C	9.709467000	7.961533000	6.014935000
H	10.031035000	7.217070000	5.271763000
H	10.292493000	8.880222000	5.855073000
N	7.771463000	3.316122000	9.608039000
I	8.303653000	-0.400304000	6.095872000
Sm	7.572405000	-3.640965000	11.367764000
O	5.229779000	-3.050081000	10.104750000
O	7.644104000	-3.269244000	8.761808000
O	6.934055000	-5.572723000	13.430251000
O	9.324381000	-5.685975000	12.005695000
O	7.503372000	-2.204836000	13.856047000
O	9.922228000	-2.324841000	12.487607000
N	5.012887000	-3.444704000	13.030756000
N	10.220804000	-3.805937000	10.014484000
C	7.592440000	2.382627000	10.302751000
N	7.341735000	-0.982957000	11.083882000
N	7.369707000	1.480463000	11.204736000
C	4.221994000	-2.427195000	10.868978000
H	3.301222000	-2.330763000	10.271930000
H	4.532588000	-1.413476000	11.161924000
C	10.011611000	-3.332216000	8.632019000
H	10.855712000	-3.632271000	7.986374000
H	9.981559000	-2.238141000	8.636221000
C	3.917115000	-3.318559000	12.057216000

H	2.993833000	-2.962693000	12.550567000
H	3.711263000	-4.315880000	11.656675000
C	5.416735000	-2.460723000	8.820228000
H	5.789399000	-1.434295000	8.921824000
H	4.460361000	-2.447719000	8.276293000
C	9.164659000	-6.421987000	13.209602000
H	9.538243000	-5.834762000	14.062582000
H	9.721306000	-7.365828000	13.151884000
C	10.590324000	-5.229690000	10.028134000
H	11.581367000	-5.393203000	9.566919000
H	9.865422000	-5.779533000	9.422383000
C	4.792746000	-4.637651000	13.868106000
H	3.719254000	-4.872699000	13.947563000
H	5.139529000	-4.436533000	14.884909000
C	6.409140000	-3.292766000	8.055368000
H	6.055678000	-4.328464000	7.960793000
H	6.549297000	-2.840202000	7.064672000
C	10.604405000	-5.851921000	11.410619000
H	10.819080000	-6.923214000	11.301462000
H	11.376160000	-5.409818000	12.059183000
C	7.368220000	0.193536000	11.053030000
C	9.681400000	-1.352982000	13.485961000
H	9.238507000	-0.450023000	13.045415000
H	10.625482000	-1.075870000	13.983184000
C	10.702707000	-1.810470000	11.412391000
H	11.535395000	-1.210281000	11.810671000
H	10.080837000	-1.161311000	10.780681000
C	6.376551000	-2.185582000	14.704583000
H	6.356567000	-1.250071000	15.285818000
H	6.423805000	-3.014849000	15.426036000
C	5.539678000	-5.853884000	13.366456000
H	5.299229000	-6.707789000	14.018209000
H	5.268530000	-6.116946000	12.334662000
C	7.707253000	-6.764613000	13.381099000
H	7.384519000	-7.393118000	12.543541000
H	7.575838000	-7.336283000	14.313423000
C	5.123318000	-2.233770000	13.850522000
H	4.245003000	-2.109479000	14.511436000
H	5.156609000	-1.369205000	13.182066000
C	8.720586000	-3.788621000	7.993734000
H	8.686899000	-3.354884000	6.985411000
H	8.634700000	-4.882442000	7.917237000
C	11.266492000	-2.986165000	10.646219000

H	11.990442000	-2.623740000	9.898785000
H	11.829564000	-3.595112000	11.357919000
C	8.728463000	-1.925815000	14.505482000
H	9.141717000	-2.837515000	14.962392000
H	8.581772000	-1.180836000	15.301833000
N	6.680641000	-5.848213000	10.257006000
C	7.227449000	-6.854089000	9.956529000
N	9.478151000	-9.091476000	10.994519000
N	7.792882000	-7.945607000	9.557839000
C	8.673959000	-8.533838000	10.342313000

1,s=0

Sm	8.069624000	5.676774000	8.412100000
Sm	7.583593000	-3.638758000	11.356005000
N	5.467896000	10.251794000	12.267260000
C	5.979479000	10.179883000	11.213469000
N	7.378482000	8.118570000	8.941531000
N	6.556954000	10.178919000	10.024361000
C	6.964476000	9.077980000	9.498714000
N	7.760132000	3.322121000	9.618619000
C	7.595220000	2.385539000	10.312717000
N	7.346203000	-0.983045000	11.080629000
N	7.392009000	1.479185000	11.215233000
C	7.379178000	0.193318000	11.056156000
N	6.691789000	-5.833571000	10.237753000
C	7.225157000	-6.848662000	9.944406000
N	9.466499000	-9.094895000	10.984306000
N	7.773878000	-7.951364000	9.554489000
C	8.659827000	-8.537696000	10.334881000
O	8.092807000	6.042452000	11.196111000
O	5.678115000	5.637759000	9.866798000
O	10.375983000	4.142255000	7.863231000
O	8.030611000	3.872546000	6.418411000
O	9.898676000	7.488260000	7.335016000
O	7.496440000	7.084336000	5.983312000
N	10.729054000	6.205354000	9.812326000
N	5.556156000	5.121618000	6.963875000
C	9.154543000	6.851167000	11.668055000
H	9.128634000	6.898721000	12.767696000
H	9.044950000	7.877418000	11.290898000
C	4.423674000	5.366189000	7.866436000
H	3.498814000	4.908821000	7.468470000
H	4.256481000	6.446869000	7.915293000

C	10.463322000	6.206555000	11.257991000
H	11.290205000	6.692031000	11.808617000
H	10.420491000	5.164572000	11.590861000
C	6.849265000	6.373860000	11.798556000
H	6.581364000	7.416421000	11.589558000
H	6.917557000	6.249067000	12.890115000
C	9.259697000	3.345613000	5.920191000
H	9.749777000	4.103905000	5.293231000
H	9.082162000	2.437781000	5.332404000
C	5.656227000	3.692141000	6.622683000
H	4.756641000	3.340429000	6.085044000
H	5.721159000	3.128638000	7.557125000
C	11.736224000	5.176788000	9.497888000
H	12.400944000	4.994982000	10.357828000
H	12.374139000	5.531060000	8.684226000
C	5.791115000	5.442921000	11.266225000
H	6.051003000	4.398398000	11.484310000
H	4.835759000	5.680816000	11.758412000
C	6.873297000	3.335240000	5.791982000
H	6.949638000	2.239523000	5.740389000
H	6.798795000	3.714219000	4.761374000
C	8.241267000	8.279561000	5.834255000
H	7.933495000	9.013639000	6.589798000
H	8.081369000	8.703630000	4.829803000
C	6.093993000	7.311221000	5.912839000
H	5.859833000	7.927535000	5.030921000
H	5.766039000	7.854060000	6.810552000
C	11.198653000	7.686167000	7.849735000
H	11.540450000	8.708437000	7.625885000
H	11.909804000	6.994192000	7.374527000
C	11.111132000	3.874028000	9.044428000
H	11.912014000	3.148322000	8.835746000
H	10.450298000	3.440230000	9.808603000
C	10.124569000	2.966227000	7.090917000
H	9.607330000	2.204873000	7.686361000
H	11.074945000	2.545173000	6.730384000
C	11.160600000	7.532403000	9.359912000
H	12.158240000	7.791681000	9.761267000
H	10.451965000	8.272770000	9.740977000
C	4.627724000	4.887180000	9.289869000
H	3.692782000	5.062756000	9.844846000
H	4.851471000	3.812454000	9.347847000
C	5.413398000	5.966046000	5.766920000

H	4.351930000	6.116724000	5.509434000
H	5.874560000	5.463526000	4.914585000
C	9.705095000	7.960467000	6.013178000
H	10.036421000	7.209093000	5.281218000
H	10.285004000	8.880288000	5.847877000
O	5.229764000	-3.038027000	10.107875000
O	7.644425000	-3.256500000	8.761398000
O	6.928506000	-5.576337000	13.423638000
O	9.318360000	-5.694252000	11.997534000
O	7.512047000	-2.210393000	13.855662000
O	9.920071000	-2.326188000	12.474323000
N	5.008266000	-3.441973000	13.037858000
N	10.227301000	-3.812030000	10.007190000
C	4.229546000	-2.409514000	10.878065000
H	3.308751000	-2.300702000	10.283116000
H	4.551179000	-1.400505000	11.175275000
C	10.013307000	-3.340712000	8.624987000
H	10.851290000	-3.648079000	7.974726000
H	9.990936000	-2.246421000	8.625375000
C	3.916197000	-3.302460000	12.063397000
H	2.994441000	-2.940051000	12.555358000
H	3.701696000	-4.296408000	11.658987000
C	5.417034000	-2.444374000	8.825595000
H	5.790474000	-1.418609000	8.930990000
H	4.460693000	-2.427997000	8.281541000
C	9.156470000	-6.432304000	13.200176000
H	9.532468000	-5.848224000	14.054286000
H	9.709984000	-7.377824000	13.140005000
C	10.593559000	-5.235978000	10.024021000
H	11.586064000	-5.402977000	9.566943000
H	9.869898000	-5.784718000	9.415792000
C	4.785768000	-4.640836000	13.864275000
H	3.712143000	-4.877310000	13.939592000
H	5.129768000	-4.448536000	14.883892000
C	6.408420000	-3.273870000	8.056088000
H	6.053426000	-4.308556000	7.956350000
H	6.549183000	-2.816693000	7.067537000
C	10.601210000	-5.857605000	11.406942000
H	10.817858000	-6.928627000	11.299023000
H	11.369364000	-5.414232000	12.058905000
C	9.688486000	-1.357787000	13.478555000
H	9.246942000	-0.451011000	13.044538000
H	10.636314000	-1.086720000	13.971793000

C	10.706216000	-1.814260000	11.401284000
H	11.537840000	-1.214934000	11.802791000
H	10.087886000	-1.164818000	10.766535000
C	6.389575000	-2.202705000	14.710981000
H	6.374205000	-1.275364000	15.305254000
H	6.441193000	-3.042230000	15.420052000
C	5.533324000	-5.853384000	13.353955000
H	5.289750000	-6.713015000	13.996964000
H	5.264241000	-6.106530000	12.319132000
C	7.697977000	-6.770373000	13.371558000
H	7.373120000	-7.396290000	12.532795000
H	7.565348000	-7.344199000	14.302403000
C	5.130190000	-2.238561000	13.865055000
H	4.257461000	-2.113465000	14.533582000
H	5.163355000	-1.369052000	13.202940000
C	8.715593000	-3.790090000	7.994579000
H	8.682432000	-3.363003000	6.983518000
H	8.619485000	-4.883548000	7.926253000
C	11.271859000	-2.991404000	10.637982000
H	11.997038000	-2.629454000	9.891325000
H	11.834073000	-3.599129000	11.351547000
C	8.739394000	-1.932372000	14.500939000
H	9.154815000	-2.844631000	14.954920000
H	8.596023000	-1.188655000	15.299159000
I	8.347355000	-0.402679000	6.100050000

2,s=5

Sm	7.889817000	5.484451000	9.214584000
O	7.535953000	7.076977000	11.476315000
O	5.351925000	5.868995000	10.317192000
O	10.372058000	4.258430000	9.372369000
O	8.212790000	3.218179000	8.054991000
O	9.701760000	6.869243000	7.611100000
O	7.586095000	5.603479000	6.440983000
N	4.552667000	10.981617000	10.216385000
N	10.293802000	6.848075000	10.460160000
N	5.713980000	4.080202000	8.071185000
C	5.193140000	10.452430000	9.388756000
N	6.986162000	7.712113000	8.549317000
N	5.912962000	9.924307000	8.412923000
C	8.493162000	8.098090000	11.684893000
H	8.314244000	8.585833000	12.654746000
H	8.403901000	8.869872000	10.907581000

C	4.423892000	4.603239000	8.544212000
H	3.603536000	3.910713000	8.284549000
H	4.231338000	5.548665000	8.027529000
C	9.859330000	7.449567000	11.730695000
H	10.599629000	8.187031000	12.089743000
H	9.802354000	6.647904000	12.473108000
C	6.217780000	7.484121000	11.817096000
H	5.887379000	8.309666000	11.174446000
H	6.193654000	7.828452000	12.861681000
C	9.539031000	2.791959000	7.727499000
H	9.911201000	3.470568000	6.951573000
H	9.526860000	1.777284000	7.310793000
C	5.922395000	2.705809000	8.562171000
H	5.134688000	2.031855000	8.180469000
H	5.843340000	2.719404000	9.650448000
C	11.396693000	5.895799000	10.724089000
H	11.863376000	6.100112000	11.698570000
H	12.180474000	6.031994000	9.973661000
C	5.304968000	6.297319000	11.668900000
H	5.611101000	5.479381000	12.334051000
H	4.281661000	6.605536000	11.930182000
C	7.278455000	2.138670000	8.156560000
H	7.618547000	1.422053000	8.913805000
H	7.249563000	1.615674000	7.191683000
C	6.446069000	8.765992000	8.533577000
C	8.218871000	6.682045000	5.776852000
H	7.735192000	7.629623000	6.046148000
H	8.162570000	6.541853000	4.686747000
C	6.223171000	5.452207000	6.062013000
H	6.142488000	5.447801000	4.965169000
H	5.639699000	6.301035000	6.446691000
C	10.932751000	7.360634000	8.103502000
H	11.277551000	8.197371000	7.477692000
H	11.699872000	6.574863000	8.060421000
C	10.935453000	4.456745000	10.663207000
H	11.802516000	3.795132000	10.801731000
H	10.181535000	4.222293000	11.426517000
C	10.387235000	2.883033000	8.966143000
H	9.981691000	2.233688000	9.752505000
H	11.421949000	2.578002000	8.747885000
C	10.717002000	7.884859000	9.509903000
H	11.643931000	8.383191000	9.847172000
H	9.935252000	8.647311000	9.454387000

C	4.379822000	4.878375000	10.031293000
H	3.377778000	5.259758000	10.281851000
H	4.558777000	3.982536000	10.642135000
C	5.743334000	4.121015000	6.595073000
H	4.749861000	3.892102000	6.177398000
H	6.430609000	3.356627000	6.229431000
C	9.664313000	6.702399000	6.202820000
H	10.173051000	5.772532000	5.910492000
H	10.169658000	7.543697000	5.707117000
N	7.758540000	4.054622000	11.047678000
I	8.543689000	-0.408745000	5.354077000
Sm	7.512020000	-3.187193000	11.124501000
O	5.721373000	-2.101907000	9.536388000
O	7.917266000	-3.325014000	8.560716000
O	6.395375000	-4.506484000	13.297705000
O	8.459241000	-5.595747000	11.867604000
O	7.569277000	-1.745200000	13.456152000
O	10.032333000	-2.687091000	12.642254000
N	5.221890000	-2.033461000	12.281486000
N	10.089697000	-4.115767000	10.135721000
C	7.067087000	3.254365000	11.709949000
N	8.453388000	-0.989267000	10.630171000
N	6.363177000	2.480184000	12.332485000
C	4.535537000	-1.463779000	9.958737000
H	3.726916000	-1.653277000	9.238059000
H	4.678706000	-0.375516000	10.017065000
C	10.282717000	-3.404181000	8.858114000
H	11.210844000	-3.735639000	8.359742000
H	10.384827000	-2.340609000	9.088697000
C	4.136478000	-2.060510000	11.292369000
H	3.247136000	-1.522256000	11.666435000
H	3.859945000	-3.105367000	11.125447000
C	6.086205000	-1.862164000	8.173544000
H	6.734347000	-0.982570000	8.098618000
H	5.186170000	-1.701876000	7.566253000
C	8.111968000	-6.166085000	13.126101000
H	8.751625000	-5.734637000	13.909352000
H	8.254953000	-7.253111000	13.097906000
C	9.877994000	-5.558121000	9.942396000
H	10.755653000	-6.051741000	9.488686000
H	9.041585000	-5.687216000	9.250246000
C	4.796920000	-2.737815000	13.502329000
H	3.738472000	-2.532060000	13.733393000

H	5.387675000	-2.377079000	14.344425000
C	6.811596000	-3.083746000	7.678870000
H	6.158392000	-3.962981000	7.687451000
H	7.174011000	-2.878246000	6.665788000
C	9.536592000	-6.281432000	11.231046000
H	9.228416000	-7.310206000	11.004494000
H	10.392827000	-6.330874000	11.918245000
C	9.060689000	0.013561000	10.501418000
C	9.954185000	-1.600175000	13.550239000
H	9.929385000	-0.650508000	12.999487000
H	10.825636000	-1.605589000	14.224957000
C	11.145025000	-2.563770000	11.768082000
H	12.066592000	-2.421666000	12.354219000
H	11.007854000	-1.688928000	11.120523000
C	6.767158000	-0.570920000	13.578302000
H	7.341028000	0.342852000	13.389743000
H	6.370117000	-0.522705000	14.603488000
C	4.999669000	-4.235685000	13.416426000
H	4.613382000	-4.698904000	14.336734000
H	4.475700000	-4.680749000	12.560174000
C	6.653487000	-5.901389000	13.397359000
H	6.037406000	-6.450806000	12.676145000
H	6.403223000	-6.258834000	14.407740000
C	5.654990000	-0.647091000	12.544840000
H	4.824626000	-0.002560000	12.871176000
H	6.038225000	-0.227097000	11.613879000
C	9.145901000	-3.564246000	7.876994000
H	9.272287000	-2.824123000	7.074457000
H	9.123763000	-4.565605000	7.423431000
C	11.256833000	-3.856504000	10.994380000
H	12.186166000	-3.851465000	10.400975000
H	11.353930000	-4.659717000	11.727183000
C	8.682107000	-1.786121000	14.335972000
H	8.683484000	-2.768546000	14.823722000
H	8.593706000	-1.015636000	15.111283000
N	5.867890000	-4.932529000	10.339180000
C	5.917916000	-6.088508000	10.085081000
N	7.267769000	-8.951518000	11.101809000
N	5.930418000	-7.331299000	9.757067000
C	6.638499000	-8.172467000	10.487574000

References

1 Bruker (2021). APEX4, SAINT and SADABS. Bruker AXS Inc., Madison, Wisconsin, USA.

2 Sheldrick, G.M. (2008) XPREP Version 2008/2. Bruker AXS Inc., Madison.

3 Sheldrick, G. M. SHELXT– Integrated space-group and crystal-structure determination. *Acta Crystallogr. Sect. A*: *Found. Adv*. **2015**, *71*, 3–8. DOI: 10.1107/S2053273314026370.

4 Sheldrick, G. M. Crystal structure refinement with SHELXL. *Acta Crystallogr., Sect. C: Struct. Chem.* **2015**, *71*, 3– 8. DOI: 10.1107/S2053229614024218.

5 Dolomanov, O. V.; Bourhis, L. J.; Gildea, R. J.; Howard, J. A. K.; Puschmann, H. OLEX2: a complete structure solution, refinement and analysis program. *J. Appl. Crystallogr*. **2009**, *42*, 339–341. DOI: 10.1107/S0021889808042726.

6 Jürgens, B.; Irran, E.; Schnick, W. Synthesis and characterization of the rare-earth dicyanamides $Ln[N(CN)_2]_3$ with Ln=La, Ce, Pr, Nd, Sm, and Eu. *J. Solid State Chem*. **2005**, *178*, 72–78. DOI: 10.1016/j.jssc.2004.10.030.

7 Wu, A.-Q.; Zheng, F.-K.; Chen, W.-T.; Cai, L.-Z.; Guo, Huang, J.-S.; Dong, Z.-C.; Takano, Y. Two Series of Novel Rare Earth Complexes with Dicyanamide $[Ln(dca)_2(phen)_2(H_2O)_3][dca]\cdot(phen)$, (Ln = Pr, Gd, and Sm) and $[Ln(dca)_3(2,2'\text{-}bipy)_2(H_2O)]_n$, (Ln = Gd, Sm, and La): Syntheses, Crystal Structures, and Magnetic Properties. *Inorg*. *Chem*. **2004**, *43*, 16, 4839–4845. DOI: 10.1021/ic035470j.

8 Nag, A.; Schmidt, P. J.; Schnick, W. Synthesis and Characterization of $Tb[N(CN)_2]_3\cdot 2H_2O$ and $Eu[N(CN)_2]_3\cdot 2H_2O$: Two New Luminescent Rare-Earth Dicyanamides. *Chem*. *Mater*. **2006**, *18*, 24, 5738–5745. DOI: 10.1021/cm0607029.

9 Tang, S.-F.; Smetana, V.; Mishra, M. K.; Kelley, S. P.; Renier, O.; Rogers, R. D.; Mudring, A.-V. Forcing Dicyanamide Coordination to f-Elements by Dissolution in Dicyanamide-

Based Ionic Liquids. *Inorg. Chem.* **2020**, *59*, 10, 7227–7237. DOI: 10.1021/acs.inorgchem.0c00667.

10 Liu, H.; Klein, W.; Bender, H.; Jansen, M. High Pressure Behavior of Mercury Cyanamide $HgCN_2$. *Z. anorg. allg. Chem.* **2002**, *628*, 1, 4–6. DOI: 10.1002/1521-3749(200201)628:1<4::AID-ZAAC4>3.0.CO;2-3.

11 Takeuchi, H.; Arai, T.; Harada, I. Structures of 18-crown-6, 15-crown-5 and their metal complexes in methanol solution as studied by Raman spectroscopy. *J. Mol. Struct.* **1986**, *146*, 197–212. DOI: 10.1016/0022-2860(86)80293-9.

12 King, S. T.; Strope, J. H. Infrared Spectra of the Argon Matrix-Isolated Cyanamide, Cyanamide-d_2, and Carbodiimide. *J. Chem. Phys.* **1971**, *54*, 1289–1295. DOI: 10.1063/1.1674967.

13 Dong, Y.; DiSalvo, F. J. Single crystal structure and Raman spectrum of $Ba_3Na_2(CN_2)_4$. *J. Solid State Chem.* **2006**, *179*, 5, 1363–1368. DOI: 10.1016/j.jssc.2006.01.048.

14 Reckeweg, O.; Schleid, T.; DiSalvo, F. J. Synthesis, Crystal Structure and Optical Spectra of $Yb_2[CN_2]_3$. *Z. Naturforsch. B.* **2007**, *62b*, 5, 658–662. DOI: 10.1515/znb-2007-0505.

15 Mączka, M.; Ptak, M.; Trzebiatowska, M.; Kucharska, E.; Hanuza, J.; Pałka, N.; Czerwińska, E. THz, Raman, IR and DFT studies of noncentrosymmetric metal dicyanamide frameworks comprising benzyltrimethylammonium cations. *Spectrochim Acta A Mol Biomol Spectrosc.* **2021**, 119416. DOI: 10.1016/j.saa.2020.119416.

16 A. D. Becke, J. Chem. Phys., 1993, 98, 5648.

17 M. Dolg, H. Stoll, A. Savin, H. Preuss, Theor. Chim. Acta 1989, 75, 173; M. Dolg, H. Stoll, H. Preuss, Theor. Chim. Acta 1993, 85, 441.

18 (a) R. Ditchfield, W. J. Hehre and J. A. Pople, J. Chem. Phys., 1971, 54, 724; (b) W. J. Hehre, R. Ditchfield and J. A. Pople, J. Chem. Phys., 1972, 56, 2257; (c) P. C. Hariharan and J. A. Pople, Theor. Chem. Acc., 1973, 28, 213; (d) J.-P. Blaudeau, M. P. McGrath, L. A. Curtiss, and L. Radom, J. Chem. Phys., 1997, 107, 5016; (e) K. Raghavachari, J. S. Binkley, R. Seeger, and J. A. Pople, J. Chem. Phys., 1980, 72, 650; (f) A. D. McLean and G. S. Chandler, J. Chem. Phys., 1980, 72, 5639.

19 Gaussian 09, Revision D.01, M. J. Frisch, G. W. Trucks,H. B.Schlegel, G. E.Scuseria, M. A. Robb, J. R.Cheeseman, G.Scalmani, V.Barone, B.Mennucci, G. A.Petersson, H.Nakatsuji, M.Caricato, X.Li, H. P. Hratchian, A. F.Izmaylov, J.Bloino, G.Zheng, J. L.Sonnenberg, M. Hada, M.Ehara, K.Toyota, R.Fukuda, J.Hasegawa, M.Ishida, T.Nakajima, Y.Honda, O.Kitao, H.Nakai, T.Vreven, J. A.Montgomery Jr., J. E.Peralta, F.Ogliaro, M.Bearpark, J. J.Heyd,E.Brothers, K. N.Kudin, V. N.Staroverov, R.Kobayashi, J.Normand, K.Raghavachari, A.Rendell, J. C.Burant, S. S.Iyengar, J.Tomasi, M.Cossi, N.Rega, M. J. Millam, M. Klene, J. E.Knox, J. B.Cross, V.Bakken, C.Adamo, J.Jaramillo, R.Gomperts, R.E.Stratmann, O.Yazyev, A. J.Austin, R.Cammi, C.Pomelli, J. W.Ochterski, R. L.Martin, K.Morokuma, V. G.Zakrzewski, G. A.Voth, P.Salvador, J. J.Dannenberg, S.Dapprich, A. D.Daniels, Ö.Farkas, J. B. Foresman, J. V. Ortiz, J. Cioslowski, D. J.Fox, Gaussian, Inc., Wallingford CT, 2009.
